# Ready-to-use therapeutic food with elevated n-3 polyunsaturated fatty acid content, with or without fish oil, to treat severe acute malnutrition: a randomized controlled trial

**DOI:** 10.1186/s12916-015-0315-6

**Published:** 2015-04-23

**Authors:** Kelsey DJ Jones, Rehema Ali, Maureen A Khasira, Dennis Odera, Annette L West, Grielof Koster, Peter Akomo, Alison WA Talbert, Victoria M Goss, Moses Ngari, Johnstone Thitiri, Said Ndoro, Miguel A Garcia Knight, Kenneth Omollo, Anne Ndungu, Musa M Mulongo, Paluku Bahwere, Greg Fegan, John O Warner, Anthony D Postle, Steve Collins, Philip C Calder, James A Berkley

**Affiliations:** KEMRI-Wellcome Trust Research Programme, Kilifi, 230-80108 Kenya; Centre for Global Health Research and Section of Paediatrics, Imperial College, Norfolk Place, London, W2 1PG UK; Faculty of Medicine, University of Southampton, Southampton General Hosptial, Tremona Road, Southampton, SO16 6YD UK; Valid Nutrition, Cuibín Farm, Derry Duff, Bantry, Co., Cork, Republic of Ireland; Southampton National Institute of Health Research Respiratory Biomedical Research Unit, Southampton General Hosptial, Tremona Road, Southampton, SO16 6YD UK; Kilifi County Hospital, Ministry of Health, Kilifi, 230-80108 Kenya; Nuffield Department of Clinical Medicine, Centre for Tropical Medicine & Global Health, University of Oxford, Old Road Campus, Roosevelt Drive, Oxford, OX3 7FZ UK; Valid International, 35 Leopold Street, Oxford, OX4 1TW UK; National Institute of Health Southampton Biomedical Research Centre, Southampton General Hosptial, Tremona Road, Southampton, SO16 6YD UK

**Keywords:** Fatty acid, Fish oils, Growth, Omega-3, Ready-to-use therapeutic food, Severe acute malnutrition

## Abstract

**Background:**

Ready-to-use therapeutic foods (RUTF) are lipid-based pastes widely used in the treatment of acute malnutrition. Current specifications for RUTF permit a high n-6 polyunsaturated fatty acid (PUFA) content and low n-3 PUFA, with no stipulated requirements for preformed long-chain n-3 PUFA. The objective of this study was to develop an RUTF with elevated short-chain n-3 PUFA and measure its impact, with and without fish oil supplementation, on children’s PUFA status during treatment of severe acute malnutrition.

**Methods:**

This randomized controlled trial in children with severe acute malnutrition in rural Kenya included 60 children aged 6 to 50 months who were randomized to receive i) RUTF with standard composition; ii) RUTF with elevated short chain n-3 PUFA; or iii) RUTF with elevated short chain n-3 PUFA plus fish oil capsules. Participants were followed-up for 3 months. The primary outcome was erythrocyte PUFA composition.

**Results:**

Erythrocyte docosahexaenoic acid (DHA) content declined from baseline in the two arms not receiving fish oil. Erythrocyte long-chain n-3 PUFA content following treatment was significantly higher for participants in the arm receiving fish oil than for those in the arms receiving RUTF with elevated short chain n-3 PUFA or standard RUTF alone: 3 months after enrolment, DHA content was 6.3% (interquartile range 6.0–7.3), 4.5% (3.9–4.9), and 3.9% (2.4–5.7) of total erythrocyte fatty acids (*P* <0.001), respectively, while eicosapentaenoic acid (EPA) content was 2.0% (1.5–2.6), 0.7% (0.6–0.8), and 0.4% (0.3–0.5) (*P* <0.001). RUTF with elevated short chain n-3 PUFA and fish oil capsules were acceptable to participants and carers, and there were no significant differences in safety outcomes.

**Conclusions:**

PUFA requirements of children with SAM are not met by current formulations of RUTF, or by an RUTF with elevated short-chain n-3 PUFA without additional preformed long-chain n-3 PUFA. Clinical and growth implications of revised formulations need to be addressed in large clinical trials.

**Trial registration:**

Clinicaltrials.gov NCT01593969. Registered 4 May 2012.

**Electronic supplementary material:**

The online version of this article (doi:10.1186/s12916-015-0315-6) contains supplementary material, which is available to authorized users.

## Background

Severe acute malnutrition (SAM) is a major risk factor for morbidity and mortality in early childhood. SAM comprises two distinct clinical syndromes, severe wasting (diagnosed on the basis of weight-for-height or mid-upper arm circumference (MUAC)) and kwashiorkor (edematous malnutrition). The global prevalence of severe wasting in children less than 5 years of age is 2.9% and, whilst the global burden of kwashiorkor is undefined, in some settings it contributes up to 50% of SAM cases [1,2]. Both syndromes are associated with greatly increased mortality from common infectious diseases such as pneumonia and diarrhea via mechanisms that are not fully understood [3,4]. Severe wasting alone underlies 7.4% of global deaths in children under five (approximately 500,000 deaths each year), and kwashiorkor is associated with a high mortality rate [1,5].

The clinical management of SAM involves a series of interventions designed to treat, ameliorate, or minimize metabolic disturbance and complications during nutritional rehabilitation. The current paradigm comprises an integrated system where inpatient care is reserved for those children who have medical complications or poor appetite, and outpatient care is provided to those who are medically stable and have appetite [6]. A key enabling factor in the implementation of community management of acute malnutrition has been the development of lipid-based ready-to-use therapeutic foods (RUTF), which aim to provide a nutritionally complete diet for rehabilitation of SAM. RUTF are microbiologically stable by virtue of their low water activity, making them suitable for use at home. The technical specification for nutritional composition of RUTF is almost identical to that for ‘F-100’ therapeutic milk, which is the standard of care for inpatient nutritional rehabilitation of children with SAM and was based, for the most part, on published specifications for infant formula manufacture [7]. There have been no major changes to the compositional specifications of F-100 or RUTF since they were originally designed [8].

The n-6 (omega-6) and n-3 (omega-3) families of polyunsaturated fatty acids (PUFA) are biologically important molecules with a wide variety of structural and functional roles. They are immunologically active, as precursors to the eicosanoid family of inflammatory mediators and as a result of their characteristic physical properties when incorporated in the lipid membranes of immune cells [9], and affect a number of risk factors for cardiovascular disease. The n-3 long-chain (LC)-PUFA docosahexaenoic acid (DHA, 22:6(n-3)) and n-6 LC-PUFA arachidonic acid (AA, 20:4(n-6)) have key structural and functional roles in the eye and brain and are thus important in early life development of these organs [10].

The short chain n-6 PUFA linoleic acid (LA, 18:2(n-6)), and the short chain n-3 PUFA alpha-linoleic acid (ALA, 18:3(n-3)) cannot be synthesized *de novo* and must be provided by dietary intake. They act as precursors for the biosynthesis of n-6 and n-3 LC-PUFA, which are produced by the sequential action of desaturase and elongase enzymes. Nevertheless, LC-PUFA may also be consumed preformed in the diet.

Specifications for F-100 and RUTF permit broad ranges of PUFA composition, with n-6 and n-3 fatty acids comprising 3 to 10% and 0.3 to 2.5% of total energy, respectively. There is no stipulation for provision of preformed LC-PUFA. LA is abundant in peanuts and many of the vegetable oils normally used in manufacturing RUTF but ALA is present at high concentration in a limited range of vegetable oils. LC-PUFA are absent from vegetable sources, meaning that RUTF prepared with only vegetable-derived lipid has low absolute n-3 PUFA and no n-3 LC-PUFA. Flax seed oil is a good source of ALA while fish oil is a good source of n-3 LC-PUFA.

The objectives of this trial were to determine whether a formulation of RUTF with elevated short chain n-3 PUFA (ALA) content is practical, safe, and acceptable in the management of children with SAM; and whether its use has an impact on children’s PUFA status, and in particular LC-PUFA status, as measured by erythrocyte fatty acid composition. In order to determine an absolute requirement for preformed n-3 LC-PUFA, the new formulation was tested with or without fish oil supplementation in addition to RUTF. Because LC-PUFA are immunologically active, secondary objectives included assessment for impact on PUFA composition and function of T cells.

## Methods

This was a single center, three-armed randomized controlled trial with balanced randomization (1:1:1). Severely acutely malnourished Kenyan children received nutritional rehabilitation with a standard RUTF (S-RUTF); a flax seed oil-containing RUTF (F-RUTF); or flax seed oil-containing RUTF with additional fish oil capsules (FFO-RUTF). Flax seed oil provides ALA while fish oil capsules provide the n-3 LC-PUFA eicosapentaenoic acid (EPA; 20:5(n-3)) and DHA. The trial was conducted double-blind between the S-RUTF and F-RUTF arms and open label with respect to FFO-RUTF.

### Participants and setting

The study took place between June 2012 and July 2013 at Kilifi County Hospital (KCH) in coastal Kenya. Kilifi County comprises a mostly rural subsistence-farming community and at least 60% of residents live below the national poverty line [11]. KCH is a government referral facility with more than 5,000 pediatric admissions per year to either a 54-bed ward or 10-bed high dependency unit where clinical care is supported by staff and funding from the Kenya Medical Research Institute (KEMRI)-Wellcome Trust Research Programme. Children with SAM are cared for in a dedicated bay where integrated nutritional and medical management is provided. Uncomplicated SAM cases are managed at KCH’s on-site outpatient therapeutic feeding program (OTP).

All children admitted to hospital and those presenting to the OTP were screened for potential eligibility. Participants were aged 6 to 60 months with SAM defined as either MUAC <11.5 cm, weight-for-height/length z-score < −3, or bilateral pedal edema (kwashiorkor), had been medically and nutritionally stabilized, and were eligible to receive RUTF according to national guidelines. Children were excluded if they were HIV-infected, undergoing treatment for tuberculosis, had other recognized or suspected major chronic inflammatory conditions (e.g., malignancy), or reported allergy or hypersensitivity to any of the product ingredients.

### Intervention

We used linear programming analysis to design a novel RUTF with increased ALA content. The RUTF was based on a standard formulation produced by Valid Nutrition (Lilongwe, Malawi) with the addition of cold-pressed flax seed oil purchased from Seed Oil SA (Somerset West, South Africa). Gas chromatographic analysis of the final recipe (performed as described later) showed that n-3 PUFA comprised 3.3% of total energy content, compared to 0.7% in the standard formulation (similar to that found in *Plumpy’nut*, the most widely available brand of RUTF; Additional file 1: Table S1), and n-6 PUFA comprised 7.9% of total energy compared to 8.2% in the standard. Both the standard formulation and the flax seed oil-containing RUTFs were packaged in identical 92 g sachets under nitrogen and stored below 25°C for the duration of the study. The two recipes were organoleptically indistinguishable. Neither recipe contained any preformed n-3 LC-PUFA. Peroxidation of the RUTF was assessed by iodometric endpoint determination (ISO 3960: 2007).

Standard or flax seed oil-containing RUTF was provided to children at a dose determined by weight according to national guidelines until MUAC was >11.5 cm, weight-for-height/length z-score > −3, or edema had resolved (depending on enrolment criteria) at two consecutive weekly visits. Parents were advised that no other foodstuff should be consumed during treatment of SAM apart from breast milk. Thereafter, RUTF was provided for use in a supplementary fashion alongside family foods at 50% of the recommended daily therapeutic dose until completion of the study, 84 days after enrolment. RUTF has been safely used in a supplementary fashion before and the recommended compositions of therapeutic and supplementary lipid-based nutritional supplements are similar [8,12]. The dose provided during the supplementary phase was often higher than recommended in national guidelines, which stipulate one 92 g sachet per day regardless of body weight. However, provision of markedly different per-kg body weight doses during the study would have introduced additional variation of intake in relation to needs and would have decreased the power of the study.

Children enrolled to the third arm of the trial were provided with two 0.5 mL fish oil capsules donated for use in the study by Seven Seas (Hull, UK), providing 214 mg of EPA plus DHA at a ratio of 1.7:1.0 (with 4 International Units vitamin E), for each 92 g sachet of (flax seed oil-containing) RUTF prescribed. Carers were instructed to pierce the capsules with a safety pin and squeeze the oil into the child’s mouth. This is the same technique used to deliver vitamin A to young children and was familiar and acceptable to participants. We calculated that participants in this arm would effectively receive 3.9% of total energy as n-3 PUFA, 16% of which would be as preformed EPA and DHA (0.39% and 0.22% total energy, respectively), compared to a DHA recommended intake for healthy infants of 0.10 to 0.18% [13]. This is likely to be an overestimate since piercing the capsule and squeezing it into the participant’s mouth was likely to preclude delivery of the entire contents.

### Study procedures

Information about the study was given to each eligible child’s attending parent or carer as soon as possible after presentation and informed consent for participation was sought. Where consent was provided, children were reviewed by a member of the study team daily, until the clinical attending team considered them medically stabilized and ready to start RUTF. At this point they were formally enrolled in the trial, given a study number (see below), and started on blinded standard or flax seed oil-containing RUTF with or without fish oil capsules according to the allocation arm. Participants who required ongoing inpatient care were reviewed by a member of the study team daily until discharge. Scheduled study follow-up took place at days 7, 14, 21, 28, 56, and 84 after enrolment. RUTF and capsules were provided at each visit. Capsules were provided in an amber plastic bottle and were dispensed by weight. Compliance was monitored by interview with the parent or carer, counting full and empty sachets of RUTF and by reweighing the returned bottles containing fish oil capsules. Percentage compliance was calculated with reference to a ‘full ration’ taking account of the participant’s weight and stage of treatment. During therapeutic feeding, additional RUTF was offered to be used after completion of the full prescribed ration if children were still hungry, in line with national guidelines. Blood samples were taken at enrolment and at days 7, 28, and 84. Monitoring for side effects or adverse events was conducted at all scheduled and unscheduled visits. Participants’ homesteads were mapped and defaulters were traced in the community.

### Outcomes

The primary outcome was erythrocyte n-3 PUFA content (percentage of the major species and n-6:n-3 PUFA content ratio) at day 84 measured by gas chromatography. Main secondary outcomes were safety and acceptability of the intervention, assessed by frequency of adverse events and compliance, respectively. The study was not powered or designed to detect differences in rate of recovery or growth, but these data were collected and are reported. A large number of other outcomes were measured in order to provide mechanistic insights relevant to future study design. Further analysis of fatty acid abundance in plasma phosphatidylcholine (at enrolment, and days 28 and 84) and T cells (at enrolment and day 84) was performed; inflammatory activation was assessed by measurement of a range of soluble mediators in plasma; insulin-like growth factor-1 (IGF-1) provided an index of linear growth potential; *in vivo* T cell activation/exhaustion and *in vitro* response to stimulation with mitogen and recall antigen were measured at enrolment and day 84; and biophysical properties of the erythrocyte membrane were assessed by response to shear stress. These outcomes were considered exploratory.

### Sample size, randomization, and blinding

Sample size was calculated with reference to changes over time in erythrocyte membrane fatty acids amongst a group of Thai schoolchildren provided with n-3 LC-PUFA-fortified milk for 6 months [14]. DHA composition of total erythrocyte fatty acids increased by 3.6% (standard deviation 1.5). We calculated the sample size based on an effect size 50% of this magnitude (because the duration of follow-up was only half as long), which came to 15 in each group. Allowing for up to 25% failure to complete the trial due to mortality or drop-out gave a final size of 20 children per arm, or 60 overall.

Standard and flax seed oil-containing RUTF were produced by Valid Nutrition (Lilongwe, Malawi). Each sachet was stamped with one of 18 indelible four-digit alphanumeric codes; 6 of the codes were designated to the standard recipe, and 12 to the flax seed oil-containing recipe (6 each for the arms with and without fish oil capsules). Access to the allocation key was restricted to manufacturers and the trial statistician (GF). A randomization list was generated in STATA (version 12.0) with variable block sizes using the following code: “*ralloc blknum blksiz Rx, nsubj(60) ntreat(3)*” [15]. The trial statistician prepared 60 opaque envelopes labeled with study numbers, inside each of which was a card identifying a four-digit RUTF code and specifying ‘with fish oil’ or ‘without fish oil’. When a participant was enrolled in the trial they were allocated the next consecutively available study number, which was entered on the allocation log prior to opening the relevant envelope.

Because of difficulties sourcing an appropriate placebo oil capsule, the FFO-RUTF arm was open label with respect to both the provision of fish oil capsules and flax seed oil-containing RUTF.

### Laboratory methods

#### Separation of blood components for analysis of fatty acid composition

Whole blood was collected into sodium heparin vacutainers (BD, Franklin Lakes, New Jersey, USA). Plasma and leukocyte fractions were isolated by separation over a discontinuous density gradient created by layering Histopaque 1077 on top of Histopaque 1119 (Sigma-Aldrich Limited, Gillingham, UK). After centrifugation at 700 *g* for 30 minutes, peripheral blood mononuclear cells were reserved, plasma was stored directly at −80°C, and the red cell pellet (free of granulocyte contamination) was washed twice in phosphate buffered saline and stored at −80°C. CD3+ T cells were isolated from peripheral blood mononuclear cells by positive selection using CD3 MicroBeads and LS columns (Miltenyi Biotec, Bergisch Gladbach, Germany) according to the manufacturer’s instructions, and also stored at −80°C.

#### Lipidomic analysis

Total lipid was extracted from the stored samples according to the method of Bligh & Dyer with dichloromethane replacing chloroform, and dried under nitrogen [16]. Plasma phosphatidylcholine was isolated using solid phase extraction on aminopropylsilica cartridges (Agilent Technologies, Santa Clara, USA). For fatty acid analysis (erythrocyte and plasma phosphatidylcholine samples), methyl esters were generated by incubation with methanol containing 2% H_2_SO_4_ and extracted into hexane following neutralization as described previously [17]. Analysis was performed on a Hewlett Packard 6890 gas chromatograph fitted with a BPX-70 column. Fatty acid methyl esters were identified using HPChemStation (Hewlett Packard, Palo Alto, USA) by retention time compared to authentic standards. For whole lipid analysis (CD3+ cells), samples were reconstituted in dichloromethane:methanol:water:concentrated ammonia (66:30:3:1) and introduced via direct infusion by nanoflow electrospray ionization to a triple quadrupole mass spectrometer (xevo-TQ, Waters, Milford, USA) [18]. Phosphatidylcholine was analyzed in positive ionization as precursors of 184+, and phosphatidylethanolamine was analyzed in the neutral loss of 141+. Individual spectra were checked in MassLynx (Waters, Milford, USA) for quality and analyzed using a custom-designed macro [19].

#### T cell activation and function

T cell activation phenotypes (CD3+, CD4/8+, CD38+, HLA-DR+ with/without PD-1 expression) were evaluated by flow cytometric analysis of fresh whole blood following staining with appropriate antibodies, and whole blood interferon gamma (IFN-γ) release in response to phytohemagglutinin (PHA) or tetanus toxoid (TT) with or without IL-12 was quantified (for further details see Additional file 1: Methods).

#### Other methods

Full blood count was performed by the Good Clinical and Laboratory Practice-compliant clinical laboratories at the KEMRI-Wellcome Trust Research Programme, Kenya. Erythrocyte deformability in response to shear stress was measured on-site using a laser-assisted optical rotation analyzer as described previously [20]. Soluble inflammatory mediators were measured in plasma (Additional file 1: Methods).

### Statistical methods

All data were double entered and validated with Openclinica (Isovera, Waltham, USA). Analysis was performed in STATA (version 12.0) and anthropometric Z-scores were calculated using World Health Organization Child Growth Standards, 2006 [21]. All analyses were done by the intention-to-treat principle. For compositional outcomes, comprising key individual molecular species, calculated total n-6 PUFA and n-3 PUFA content, and n-6:n-3 ratios, analysis of variance (ANOVA) models were used to test differences between the three arms at each time point, and Mann–Whitney U-tests to identify within-arm changes from baseline. Compliance was calculated and compared between arms using ANOVA. For grouped variables (e.g., adverse events), significance was calculated using Fisher’s exact test, or χ^2^ test where the expected number in all cells was >5. Changes in anthropometric indices were calculated for individual participants and compared between groups using ANOVA. Where indicated in the text, S-RUTF and F-RUTF arms were considered together as both not containing fish oil, and F-RUTF and FFO-RUTF were considered together as both containing flax seed oil. We performed a *post hoc* analysis to assess for impact of baseline erythrocyte fatty acid composition on changes in erythrocyte membrane composition over the trial using linear regression and assessing for effect modification on the regression coefficient by arm. ‘Long-chain’ is used to indicate 20-carbon or longer chain fatty acids throughout.

### Study oversight

All participants enrolled in the study had individual written informed consent provided by a parent or guardian. The study was approved by the KEMRI Ethical Review Committee and the Oxford Tropical Research Ethics Committee prior to initiation. The University of Oxford was the sponsor. Clinical trials monitoring was performed by staff from the Clinical Trials Facility at the KEMRI-Wellcome Trust Research Programme. An independent trial steering committee acted as the decision-making body for the study and an independent pediatrician acted as local safety monitor. Neither the sponsor nor any other party except the named investigators had any role in the design of the study, interpretation of the results, content of manuscripts, or decision to publish. The trial was registered at https://clinicaltrials.gov/ct2/show/NCT01593969.

## Results

**Figure 1:**
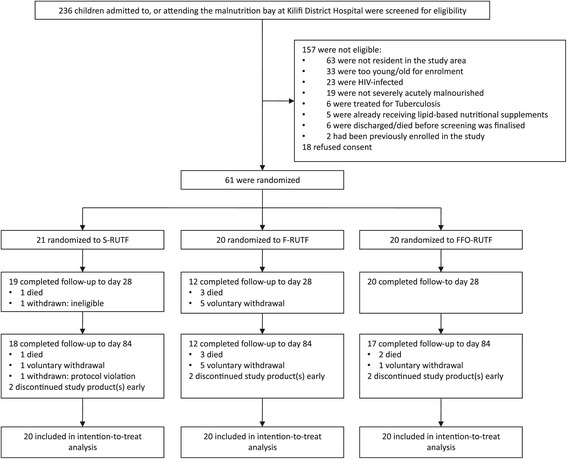
**Trial flow diagram.**

Between May 30, 2012, and April 30, 2013, 236 children admitted to, or attending, the OTP and inpatient malnutrition bay at KCH were assessed for eligibility; 61 children were enrolled in the trial, one of whom was withdrawn as ineligible (suspected hematological malignancy) within 24 hours of enrolment and is not included in any of the analyses (Figure 1).

### Stability of the RUTF

Twelve months after manufacture (May 9, 2012) of the two batches of RUTF we performed a precautionary reanalysis of the lipid composition of the flax seed oil-containing formulation to check for deterioration. Although fatty acid composition (by gas chromatography) and organoleptic qualities were unchanged, on April 18, 2013, the peroxide content of the flax seed oil-containing product was 17.9 meq/kg, which is higher than the value stipulated by UNICEF as acceptable for newly manufactured batches (<10 meq/kg). Because deterioration of RUTF can occur rapidly once peroxidation begins, we began regular checks on palatability and peroxide levels. On May 16, 2013, the peroxide levels had risen to 33.5 meq/kg, and investigators considered the flax seed oil-containing product had become less palatable (despite the fact that peroxide levels were 29.7 meq/kg in the standard formulation, it remained palatable). In discussion with the independent trial steering committee, provision of all study RUTF and capsules was stopped. Children who still required therapeutic or supplementary feeds were switched over to the hospital’s standard supply. Six children were affected, 2 in each arm. There were no adverse events considered attributable to the deterioration and these 6 participants were included in intention-to-treat analyses.

### Baseline characteristics

The arms were comparable at baseline. Children allocated to FFO-RUTF tended to have lower MUAC (*P* = 0.08) and were less likely to have diarrhea at presentation (*P* = 0.12) (Table 1). There were no major differences in baseline fatty acid composition of any of the compartments tested, or any of the inflammatory or hematological indices.

**Table 1:** **Baseline characteristics of the participants**

**Baseline characteristics**	**S-RUTF**	**F-RUTF**	**FFO-RUTF**
Participants^a^	20	20	20
Age, months	18 (9 to 30)	16 (11 to 25)	14 (11 to 21)
Sex: Male	12 (60)	10 (50)	11 (55)
***Nutritional Status***			
Mid-upper arm circumference, cm	11.2 (10.9 to 11.4)	11.3 (10.8 to 11.9)	10.9 (10.4 to 11.4)
Weight-for-height/length z score	−3.11 (−4.43 to −1.89)	−3.31 (−3.88 to −2.90)	−3.65 (−4.12 to −3.19)
Bilateral pedal edema (kwashiorkor)	1 (5)	1 (5)	0 (0)
Height-for-age z score	−3.11 (−3.90 to −1.93)	−3.36 (−4.58 to −2.55)	−2.58 (−4.20 to −1.97)
Head circumference-for-age z score	−1.38 (−3.36 to −0.62)	−1.85 (−2.82 to −1.02)	−1.37 (−2.27 to −0.56)
Breastfeeding	8 (40)	8 (40)	12 (60)
Complementary feeds introduced, months of age	5 (2 to 6)	4 (2 to 6)	4 (3 to 6)
***Complications***			
Diarrhea	11 (55)	8 (40)	4 (20)
Pneumonia	3 (15)	3 (15)	1 (5)
Shock	1 (5)	2 (10)	0 (0)
Congenital heart disease	3 (15)	1 (5)	3 (15)
Cerebral palsy	3 (15)	1 (5)	4 (20)
***Demographics***			
Main carer is not the mother	4 (20)	5 (25)	1 (5)
Main carer has no primary education	7 (35)	8 (40)	8 (40)
Lives in mud-walled house	13 (65)	15 (75)	17 (85)
No toilet in house/compound	8 (40)	6 (30)	5 (25)
Fisherman in household	1 (5)	2 (10)	2 (10)

Data are number (%) or median (inter-quartile range) unless otherwise specified.

^a^Excluding one child withdrawn due to ineligibility (S-RUTF arm).

S-RUTF, Trial arm receiving standard-formulation RUTF without fish oil capsules; FFO-RUTF, Trial arm receiving flax seed oil-containing RUTF and fish oil capsules; F-RUTF, Trial arm receiving flax seed oil-containing RUTF without fish oil capsules.

### Follow-up and compliance

Seven children were voluntarily withdrawn at parental request: 1 in the S-RUTF arm (at day 84), 5 in the F-RUTF arm (4 before day 7, 1 at day 28), and 1 in the FFO-RUTF arm (at day 42; *P* = 0.41 between both elevated n-3 PUFA (F-RUTF and FFO-RUTF) and the standard RUTF arms). Compliance with RUTF feeding and capsules amongst children still in follow-up was high in all three arms (Table 2).

**Table 2:** **Compliance, safety, and growth**

**Compliance**	**S-RUTF**	**F-RUTF**	**FFO-RUTF**	***P***
% RUTF to d28	89 (62 to 102)	95 (79 to 102)	95 (88 to 103)	0.42
% RUTF to d84	90 (80 to 101)	96 (67 to 100)	92 (76 to 100)	0.98
% Capsules to d28	–	–	90 (71 to 99)	–
% Capsules to d84	–	–	88 (79 to 95)	–
**Safety**				
Deaths	1	3	2	0.86
Other serious adverse events	5	0	4	–
Total death or serious adverse event	6	3	6	0.63
Total illness episodes	50	47	54	0.27
**Common illness syndromes**				
Upper respiratory tract infection	7	19	16	0.08
Lower respiratory tract infection	3	2	4	0.90
Rash	10	5	13	0.16
Diarrhea	7	7	4	0.75
Vomiting	2	6	7	0.20
**Growth**				
*Mid-upper arm circumference change*				
Enrolment to d28 (mm/day x10^3^)	46 (21 to 72)	52 (24 to 68)	54 (36 to 75)	0.53
Enrolment to d84 (mm/day x10^3^)	21 (17 to 39)	21 (17 to 29)	21 (18 to 29)	0.48
*Weight-for-height/length change*				
Enrolment to d28 (z score/day x10^3^)	57 (21 to 80)	60 (47 to 84)	58 (34 to 88)	0.23
Enrolment to d84 (z score/day x10^3^)	20 (14 to 35)	21 (14 to 33)	23 (15 to 27)	0.96
*Height/length-for-age change*				
Enrolment to d28 (z score/day x10^3^)	−11.9 (−22.9 to −2.5)	−8.0 (−20.9 to −1.0)	−10.4 (−15.7 to 2.9)	0.75
Enrolment to d84 (z score/day x10^3^)	3.0 (−4.5 to 5.6)	4.0 (0.4 to 5.8)	0.2 (−2.3 to 4.6)	0.40
*Head circumference-for-age change*				
Enrolment to d28 (z score/day x10^3^)	10.1 (1.8 to 17.1)	13.2 (5.8 to 17.0)	6.1 (−6.4 to 20.4)	0.71
Enrolment to d84 (z score/day x10^3^)	8.1 (0.6 to 9.8)	5.8 (3.9 to 8.4)	7.4 (1.2 to 9.3)	0.96

Data are absolute numbers or median (inter-quartile range) unless otherwise specified. Total illness episodes are compared by χ^2^ test. Individual illness syndromes, deaths, and deaths/serious adverse events are by Fishers Exact test. Compliance and growth by ANOVA.

S-RUTF, Trial arm receiving standard-formulation RUTF without fish oil capsules; FFO-RUTF, Trial arm receiving flax seed oil-containing RUTF and fish oil capsules; F-RUTF, Trial arm receiving flax seed oil-containing RUTF without fish oil capsules.

### Safety

Six children (10%) died during follow-up; 5 of these deaths were associated with severe pneumonia and 1 occurred in the community where we were unable to ascertain cause of death. One death occurred in the S-RUTF arm, 3 in the F-RUTF, and 2 in the FFO-RUTF arms. There were a further 9 severe adverse events (requiring hospitalization or prolonging hospital stay): 4 were lower respiratory tract infection (2 in S-RUTF, 2 in FFO-RUTF), 4 were diarrhea/dehydration (3 in S-RUTF, 1 in FFO-RUTF), and 1 was severe malaria (FFO-RUTF). There were no adverse events considered directly attributable to the investigational products.

Non-severe episodes of illness or infection were common during follow-up, as expected in this high-risk population. Total number of illness episodes and the nature of the episodes were similar between the groups (Table 2). There was a greater number of upper respiratory tract infections and of vomiting episodes reported amongst children receiving the RUTF with elevated n-3 PUFA (F-RUTF and FFO-RUTF arms), but neither of these effects were statistically significant.

### Lipidomic analysis

#### Erythrocytes

**Figure 2:**
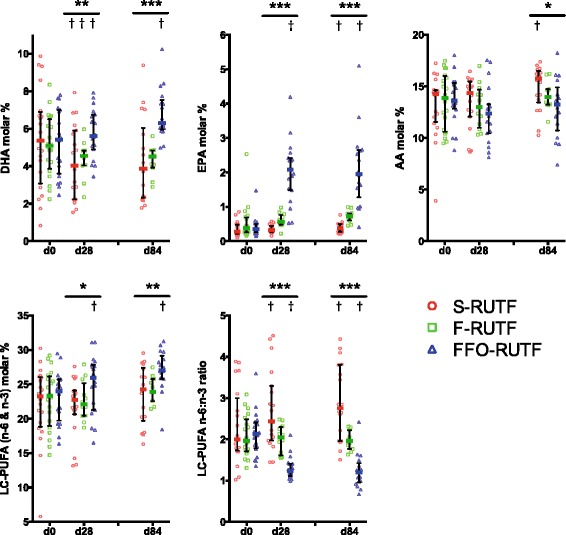
**Erythrocyte fatty acid composition.** Median and interquartile ranges shown for the three arms at baseline, day 28, and day 84. Graphs show (clockwise from top left) DHA, EPA, AA, n-6:n-3 ratio in LC-PUFA, and total LC-PUFA (n-6 and n-3). For between-arm comparisons (ANOVA) at each time point: *P ≤0.05; **P ≤0.01; ***P ≤0.001. For within-arm comparison (sign test) to baseline values: † *P* ≤0.05. LC indicates species with carbon chain >18.

There were large and highly significant differences in erythrocyte membrane fatty acid composition between the groups at days 28 and 84 (Figure 2 and Additional file 1: Table S2). The percentage of PUFA increased at the expense of saturated fatty acids, but there were wide differences in the behavior of individual species. In the S-RUTF arm there were increases in n-6 PUFA family members dihomo-gamma-linolenic acid (20:3(n-6)) and AA, and also in EPA, but DHA declined significantly during follow-up. Although breastfeeding was an important determinant of baseline DHA status, the decline in DHA occurred in both breastfed and non-breastfed children (Additional file 1: Figure S1). In the FFO-RUTF arm, there were marked increases in most of the n-3 LC-PUFA species (except eicosatetraenoic acid (20:4(n-3))), while n-6 PUFA family members were unchanged from baseline. Compared to the other two arms, erythrocyte composition in the F-RUTF arm was least affected, with modest increases in EPA and a decline in DHA similar to that seen in S-RUTF.

Alteration in the LC-PUFA species lay behind significant changes in the overall erythrocyte n-6:n-3 PUFA ratio, but despite the provision of diets with dramatically different LA and ALA content there was no difference in the LA:ALA ratio between the groups at any timepoint, and no change from baseline.

Ratios of 20:4(n-6)/20:3(n-6), 18:3(n-6)/18:2(n-6), and 20:3(n-6)/18:3(n-6) were calculated as indices of ∆5-desaturase, ∆6-desaturase, and elongase activity, respectively. There were no differences between the arms at any timepoint, and no evidence suggesting longitudinal change in enzyme activity during nutritional rehabilitation (Additional file 1: Figure S2).

**Figure 3:**
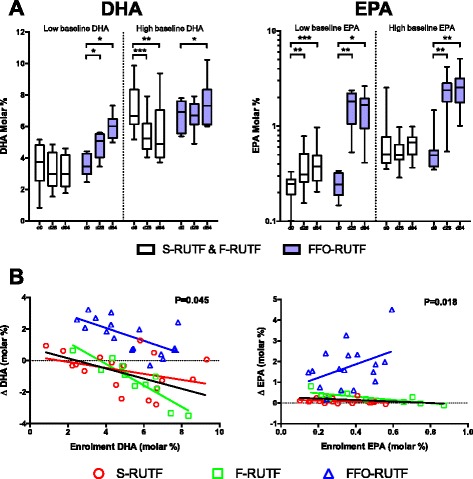
**Effect modification of baseline PUFA status on EPA and DHA enrichment in erythrocytes. (A)** Erythrocyte DHA and EPA between both arms without fish oil (S-RUTF and F-RUTF) compared to FFO-RUTF. Participants are stratified on the basis of their baseline DHA or EPA values. Low indicates equal to or below the median, and high indicates above the median. *P ≤0.05; **P ≤0.01; ***P ≤0.001 (by sign test). **(B)** Change in erythrocyte DHA or EPA from baseline to day 84 plotted against baseline DHA or EPA. Regression lines are colored the same as corresponding group symbols, black regression line is for S-RUTF and F-RUTF combined. *P* value is for effect modification across the three arms as described in the text.

Baseline composition of LC-PUFA was an important modulator of the compositional response to PUFA provision. In the FFO-RUTF arm, children with low baseline values of DHA had large increases, whilst amongst those with relatively higher baseline DHA, enrichment was much less marked (Figure 3A). For those in both of the non-fish oil arms, relatively low baseline DHA remained constant; however, there was a marked decrease in DHA amongst children who had higher levels at baseline. The regression coefficients for the change in DHA by baseline composition differed between the trial arms (*P* = 0.045 overall, and *P* = 0.025 between the S-RUTF and F-RUTF arms; Figure 3B).

For EPA, the situation was different. Fish oil supplementation was associated with large increases in erythrocyte EPA content regardless of baseline status, and for those in the two non-fish oil arms, only those with relatively low baseline EPA levels had any appreciable relative increase during the study. The regression coefficients for the change in EPA by baseline composition were significantly different between arms (*P* = 0.018 overall, and *P* = 0.032 between FFO-RUTF and F-RUTF arms). The fact that increases in EPA composition were largest in those children in the fish oil group who had highest levels at baseline, implies that provision of preformed EPA bypassed regulatory mechanisms (Figure 3B).

#### Plasma phosphatidylcholine

Average changes in percentage composition of plasma phosphatidylcholine PUFA were very similar to those seen in the erythrocyte fraction, although plasma phosphatidylcholine was less enriched in PUFA overall and the observed effect sizes were smaller (Additional file 1: Table S3). However, the relationships between participant-level erythrocyte and plasma phosphatidylcholine data were highly variable (Additional file 1: Figure S3 and Table S4).

#### T cells

Differences in the T cell compartment were less marked. Modest relative increases in AA in the S-RUTF arm and in EPA in the FFO-RUTF arm were evident in phosphatidylcholine species, but DHA was unaffected. There was no effect of the intervention on phosphatidylethanolamine species (where EPA was undetectable) (Additional file 1: Table S5 and S6).

### Growth

There were no detectable differences between the arms in any of the growth indices measured (Table 2). There was no difference in IGF-1 between the arms at any timepoint but all arms had a highly significant increase in IGF-1 by day 28 compared to baseline, which was sustained to day 84 (Additional file 1: Table S7).

### Hematologic indices

Hemoglobin increased over the course of the trial in all arms, and there were no differences between the arms in any of the hematologic indices measured (including red cell deformability) (Additional file 1: Figure S4). Marked thrombocytosis developed over the early part of the study in some participants. There was no clinical evidence of coagulopathy in any participant at any point in the trial.

### Inflammatory indices

There were no major differences in soluble inflammatory markers in plasma between the arms at any time point (Additional file 1: Table S7). There was a general reduction in levels of both pro- (e.g., IL-8, CXCL10) and anti-inflammatory (e.g., IL-10) cytokines during the course of the study. There were no differences in the proportion of activated (CD38, HLA-DR co-expressing with/without PD-1 expression) CD4 or CD8 T cells between groups at day 84, though the proportion of PD-1 co-expressing CD8 T cells had fallen in the S-RUTF and F-RUTF arms (Additional file 1: Table S8). There was no significant difference between the arms in IFN-γ release upon stimulation with PHA with or without IL-12, or TT with IL-12, at enrolment or day 84. Modest increases in IFN-γ release in both of the PHA stimulation conditions were most pronounced in the F-RUTF and FFO-RUTF arms. IFN-γ release induced by TT without IL-12 was undetectable in cultures from most participants (Additional file 1: Table S8). There were no significant associations between any of the T cell functional parameters and corresponding T cell LC-PUFA compositional indices.

## Discussion

This study has shown that treatment of SAM with conventional RUTF is associated with a decline in DHA status. The production and administration of RUTF with elevated n-3 PUFA (as ALA), with or without additional fish oil as a supplement, to children with SAM is technically feasible, acceptable to patients and their carers, and safe: the 10% mortality rate was in line with our and others’ experience treating this extremely vulnerable population with complicated SAM [22-25]. Provision of RUTF with elevated ALA had minimal impact on n-6 and n-3 PUFA status, but addition of fish oil was associated with marked increases in n-3 LC-PUFA across multiple compartments. Importantly, in both arms without fish oil, the percentage composition of DHA in erythrocytes declined, suggesting an absolute requirement for preformed DHA in the nutritional management of SAM.

An essential aim in the design of RUTF is to provide optimal nutrient intake for growth and development of somatic and neural tissues. RUTF compositional specifications were based on infant formula guidelines, but in the period since they were first developed a new consensus has emerged that preformed LC-PUFA should be added to infant formula in view of the fact that formula-fed infants’ erythrocyte DHA declines without such exogenous provision [26]. Dietary supply of DHA is considered conditionally essential for infants and young children [13]. Our data have shown that similar declines occur during nutritional rehabilitation of SAM using RUTF according to current compositional guidelines. The impact of small relative decreases in erythrocyte DHA content in this context remains unclear. However, DHA is a major component of neural lipid, and deficiency during early childhood has been linked to a range of neurodevelopmental abnormalities [27]. Children with SAM are at risk of long-term cognitive and behavioral deficits [28], and it is plausible that inadequate DHA provision during nutritional rehabilitation could be an exacerbating factor. Because routinely providing preformed n-3 LC-PUFA to children with SAM may have substantial resource and practical implications, policy on formulation should be based on a clear assessment of clinical (especially neurodevelopmental) utility, and trials evaluating these outcomes should be prioritized. In 2011, RUTF was provided to 1.96 million children, fewer than 10% of the many millions who needed it [29]. Concerns around its composition should not detract from the clear current imperative to support and expand coverage.

ALA can theoretically act as a substrate for biosynthesis of all the longer chain n-3 PUFA family members, but participants in the F-RUTF arm, who received far more ALA than those in the S-RUTF arm, had relatively modest changes in n-3 LC-PUFA after three months’ treatment. Point estimates for percentage content of EPA and docosopentaenoic acid (DPA, 22:5(n-3)) were increased, but the failure to impact positively on DHA was notable. The results bear comparison to previous clinical studies, which have suggested that while provision of ALA can be shown to drive increases in EPA and DPA content, metabolic conversion all the way to DHA is inefficient, and a dietary supply of preformed DHA is conditionally essential for its tissue enrichment [30-32]. Recent experimental work suggests that provision of large quantities of dietary ALA might inadvertently further limit its already slow metabolism to DHA. By systematically varying LA and ALA intake in rats, Gibson et al. found that increasing ALA above an optimal concentration was independently associated with a decrease in tissue DHA accumulation due to competitive inhibition of a part of the conversion step from DPA to DHA catalyzed by ∆6 desaturase [33]. Similar inhibition may occur by competition for elongase enzyme activity [34]. It is therefore possible that although by providing elevated ALA content we increased the available substrate for DHA biosynthesis, ALA may have inhibited those same metabolic pathways to which it is subject, rendering the supplementation futile as regards DHA accretion. Although it is reassuring that children in the arm receiving elevated ALA without fish oil did not see a decrease in DHA levels beyond that seen with the standard RUTF formulation, this theoretical concern means that we do not believe that RUTF formulations with high ALA content should be taken forward to further clinical trials. Furthermore, although desaturase and elongase activity was similar between the arms, the fact that AA differed after three months’ follow-up is difficult to explain by any means other than an ALA-dependent decrease in LA conversion to AA. This alone might have had important consequences, since AA status has been related to growth [35].

An alternative strategy to improve DHA accretion during nutritional rehabilitation may be to reduce the n-6 PUFA content of RUTF, because it is well recognized that n-6 PUFA can interfere with desaturation and elongation of ALA [36]. An attractive feature of such an approach is the possible avoidance of problems associated with stability during storage for products with increased PUFA content. Although we believe that the high drop-out rate in the F-RUTF arm is likely to be random statistical noise (it did not occur in FFO-RUTF arm, who also received the flax seed oil-containing product), the fact that the flax seed oil-containing RUTF became unusable after just a year despite packaging under nitrogen and storage below 25°C presents a major practical impediment to its use in countries or areas with limited resources. There are precedents for n-6 PUFA reduction as a means of increasing n-3 LC-PUFA biosynthesis, although the effectiveness in terms of DHA accumulation in clinical studies has been modest to date [37-39]. An important consideration will be to ensure that n-6 LC-PUFA status itself is not compromised, since this may have adverse consequences for growth, as noted previously [35]. Notwithstanding these concerns, a major part of the reason that RUTF have been successful is that it is straightforward to administer, and the development of a formulation that could address DHA accretion without resorting to provision of encapsulated fish oil should be the eventual aim. In pilot work (unpublished), we attempted to synthesize an RUTF containing fish oil as part of the RUTF mix, but it quickly became rancid. There is considerable interest in the production of n-3 LC-PUFA-based products that are resistant to environmental oxidation, but achieving durable stability as part of a mix with highly oxidizing micronutrients, such as iron, will be a major challenge. On the other hand, a recent paper describing the results of a clinical trial of using RUTF with different PUFA compositions in the treatment of SAM in Malawi, has suggested that even higher doses of ALA than we employed might have benefit [40]. In this study, treatment with an RUTF with very-high LA (21.3% of total fatty acids) and low ALA (0.4%) was associated with a decrease in DHA content of plasma phospholipids after four weeks, but a modified product with lower LA content (although, at 13.1%, still similar to Plumpy’nut) and high ALA (13.1%) protected against this decline. There was no evidence of a detrimental impact on growth or recovery from SAM; in fact, children in the modified RUTF arm experienced a greater improvement in weight-for-height than those in the standard arm, although the high prevalence of kwashiorkor at baseline means that this result is difficult to interpret. The modified formulation contained ALA at more than twice the level in our flax seed oil-enriched formulation. Our experience suggests that achieving long-term stability of a preparation with such high ALA content may be difficult, but if stability could be achieved, then this formulation might minimize DHA depletion whilst maintaining the practicality of an exclusively RUTF-based regime.

Although there is no precise cutoff for defining DHA deficiency, Luxwolda et al. demonstrated that transplacental materno-fetal transfer of DHA is actively regulated towards providing infants with 5.9% DHA composition in erythrocytes at birth, and it is hypothesized that DHA composition of around 7% is optimal for prevention of some non-communicable diseases in adulthood [41-43]. By comparison, children enrolled in this study had marginally low baseline DHA (median (interquartile range), 5.2% (3.7–6.7)), but regulated their erythrocyte composition at around 7% in the presence of preformed DHA in the diet. Several previous studies have investigated fatty acid status in malnourished children, and while analyses of erythrocyte and plasma lipids have proved highly inconsistent, relatively low levels of AA and DHA are most frequently reported [44-51]. Composition of plasma and erythrocytes should only be regarded as proxy measures of sufficiency, since it is possible that preservation of their composition is achieved by diversion away from other tissue compartments. There is little doubt that children with SAM are at high risk for having very low recent n-3 LC-PUFA intake. For young children, the major dietary source of n-3 and n-6 PUFA is breast milk, the composition of which is critically dependent on maternal intake [52]. A systematic review of studies measuring breast milk composition revealed that breast milk from sub-Saharan African mothers has consistently high AA content but very variable DHA content, probably driven by large local discrepancies in access to fish [53]. Access to dietary sources of fish and n-3 PUFA is strongly related to GDP on a per-country basis [54], and a detailed analysis of fatty acid intake by Gambian children revealed a steep decline in n-3 PUFA and preformed LC-PUFA at the point of weaning [55]. In conditions of moderate or intermittent food insecurity far from sources of affordable fish (where intake of n-3 LC-PUFA is likely to be marginal) it is reasonable to assume that reduced food security would be associated with reduced fish intake for both breastfeeding mothers and their children in many circumstances.

While DHA content appeared to be regulated, EPA did not – the greatest increases in erythrocyte EPA with fish oil provision being among those participants with the highest baseline values (Figure 3B). Percentage EPA composition reached levels equivalent to those seen in populations that consume very large amounts of marine fish [56]. It is plausible that providing preformed EPA (but not, apparently, DHA) bypasses a physiologically beneficial regulatory checkpoint, and detailed assessment of safety outcomes should form an important component of future studies if oils containing high levels of EPA, as used here, are employed. We deliberately chose to use fish oil with high EPA content because we hypothesized that its anti-inflammatory properties might be beneficial in the context of SAM. The lack of any measurable impact on inflammation in this study further discourages the use of such oils in future work. Understanding the kinetics of EPA accumulation would be helped by fatty acid desaturase gene cluster profiling, given that common polymorphisms could have a substantial impact on PUFA metabolic responses to supplementation [57]. This should be considered as a component of further research.

This study builds on previous work in different settings. Smit et al. randomized 17 Pakistani children with low weight-for-age z-score to 500 mg/day of fish oil alongside standard care (n = 10), which consisted of multivitamin provision and parental nutritional education, or standard care alone for up to 12 weeks [58]. They demonstrated a marked increase in erythrocyte composition of all n-3 LC-PUFA in the intervention arm but no change from baseline in the controls. Koletzko et al. demonstrated an increase in n-3 LC-PUFA in plasma phospholipids from baseline in a cohort of 8 Nigerian children with SAM after two weeks of follow-up when provided a rehabilitation diet that contained fish [45]. In a recent controlled trial of fish oil supplementation for young infants without SAM in the Gambia, van der Merwe et al*.* effected increases in plasma n-3 LC-PUFA and in MUAC in the intervention group [59]. There was no difference in intestinal health, frequency of illness, or neurocognitive development, but breastfeeding rates were high in the trial and the mothers’ breast milk was unusually rich in DHA, meaning that the participants were much more likely to be n-3 LC-PUFA replete than those in our study.

n-6 and n-3 LC-PUFA and their metabolites are potent immunomodulatory agents, with n-6 PUFA family members being broadly pro-inflammatory and n-3, anti-inflammatory [9]. Children with SAM have a chronic inflammatory T cell-associated enteropathy (environmental enteric dysfunction), which may be partly maladaptive and disrupt beneficial effects of nutritional rehabilitation [60]. Provision of RUTF with a high n-6 PUFA content could exacerbate this inflammatory activation, thereby further disturbing mucosal homeostasis and contributing to worsening of gastrointestinal symptoms, increase in microbial translocation, and persistent growth failure. However, although we demonstrated major differences in n-3 PUFA composition between the three arms in multiple compartments, there was no evidence of consistent or major impact on any of the numerous immunologic and inflammatory indices measured in exploratory analyses. Hospitalized children with SAM are an extremely heterogeneous group. Some have or are recovering from a major infection, others have chronic illnesses, and some present early without major medical or metabolic complications, but require a short period of hospital care because of inadequate facilities for care at home. A wide range of inflammatory and immunologic status would therefore be expected at baseline and the impossibility of controlling or correcting for this in a small trial render the likelihood of a type 2 error high. Additionally, while understanding of the importance of PUFA in immune function is informed by a wealth of data from model and experimental settings encompassing a wide range of methodological approaches, our cellular and molecular understanding of the functionally immunocompromised state associated with SAM is extremely limited [4,9]. Our choice of assays was hypothesis-based but evidence underlying these hypotheses is weak. Further careful observational work to determine the relationship between enteric inflammation, mucosal and systemic immune function, and nutritional status is a research priority, and the utilization of exploratory and systems biological approaches are likely to be useful in challenging established models.

## Conclusions

PUFA requirements of children with SAM are not met by RUTF manufactured according to current specifications, and are associated with a drop in DHA during nutritional rehabiliation. Although this trial was well powered to assess for compositional indices as primary outcome, it was not designed or powered to assess for clinically important secondary outcomes such as growth and frequency of infectious episodes. The safety and acceptability of the approach employed provides a sound foundation for future trials targeting such outcomes on a much larger scale. That our RUTF formulation with elevated ALA content did not enrich for DHA and quickly became unusable, suggests that simply increasing the stipulated ALA content of RUTF is not a sufficient adaptation to current standards, and such a formulation is not a rational one to take forward for larger-scale trials. Low n-6 PUFA content formulations are potential candidates, but for trials designed to show proof of concept that raising n-3 LC-PUFA has clinical and growth benefits, providing the preformed molecules themselves may provide the clearest, quickest, and most unambiguous answer.

## Additional file

Additional file 1:
**Supplementary Information.**

## Abbreviations

AA: Arachidonic acid
ALA: Alpha-linolenic acid
ANOVA: Analysis of variance
DHA: Docosahexaenoic acid
DPA: Docosapentaenoic acid
EPA: Eicosapentaenoic acid
FFO-RUTF: Trial arm receiving flax seed oil-containing RUTF and fish oil capsules
F-RUTF: Trial arm receiving flax seed oil-containing RUTF without fish oil capsules
IFN-γ: Interferon gamma
IGF-1: Insulin-like growth factor-1
KCH: Kilifi County Hospital
KEMRI: Kenya Medical Research Institute
LA: Linoleic acid
LC-PUFA: Long-chain polyunsaturated fatty acid
MUAC: Mid-upper arm circumference
OTP: Outpatient therapeutic feeding programme
PHA: Phytohemagglutinin
PUFA: Polyunsaturated fatty acid
RUTF: Ready-to-use therapeutic food
SAM: Severe acute malnutrition
S-RUTF: Trial arm receiving standard-formulation RUTF without fish oil capsules
TT: Tetanus toxoid

## References

[1] Black RE, Victora CG, Walker SP, Bhutta ZA, Christian P, de Onis M (2013). Maternal and child undernutrition and overweight in low-income and middle-income countries. Lancet.

[2] Briend A, Myatt M, Dent N, Brown R. Putting kwashiorkor on the map. CMAM Forum 2013. http://www.cmamforum.org/Pool/Resources/Putting-kwashiorkor-on-the-map-CMAM-Forum-2013.pdf.

[3] Black RE, Allen LH, Bhutta ZA, Caulfield LE, de Onis M, Ezzati M (2008). Maternal and child undernutrition: global and regional exposures and health consequences. Lancet.

[4] Rytter MJ, Kolte L, Briend A, Friis H, Christensen VB (2014). The immune system in children with malnutrition–a systematic review. PLoS One.

[5] Bejon P, Mohammed S, Mwangi I, Atkinson SH, Osier F, Peshu N (2008). Fraction of all hospital admissions and deaths attributable to malnutrition among children in rural Kenya. Am J Clin Nutr.

[6] World Health Organization, World Food Programme, United Nations System Standing Committee on Nutrition, United Nations Children’s Fund. Community-based management of severe acute malnutrition: a joint statement. Geneva: WHO; 2007. http://www.who.int/nutrition/topics/Statement_community_based_man_sev_acute_mal_eng.pdf?ua=1.

[7] United Nations Development Programme – Inter-Agency Procurement Support Office. Emergency relief items – compendium of generic specifications, volume 1. Copenhagen: UNDP; 1995.

[8] UNICEF. Supply Catalogue. 2014. https://supply.unicef.org/.

[9] Calder PC (2013). n-3 fatty acids, inflammation and immunity: new mechanisms to explain old actions. Proc Nutr Soc.

[10] Calder PC (2014). Very long chain omega-3 (n-3) fatty acids and human health. Eur J Lipid Sci Technol.

[11] Kenya Open Data. Poverty rate by district. https://opendata.go.ke/Poverty/Poverty-Rate-by-District/i5bp-z9aq.

[12] Nackers F, Broillet F, Oumarou D, Djibo A, Gaboulaud V, Guerin PJ (2010). Effectiveness of ready-to-use therapeutic food compared to a corn/soy-blend-based pre-mix for the treatment of childhood moderate acute malnutrition in Niger. J Trop Pediatr.

[13] Food and Agriculture Organization of the United Nations. Fats and fatty acids in human nutrition: report of an expert consultation, FAO Food and Nutrition Paper, vol. 91. Rome: FAO; 2010. 21812367

[14] Thienprasert A, Samuhaseneetoo S, Popplestone K, West AL, Miles EA, Calder PC (2009). Fish oil n-3 polyunsaturated fatty acids selectively affect plasma cytokines and decrease illness in Thai schoolchildren: a randomized, double-blind, placebo-controlled intervention trial. J Pediatr.

[15] Ryan P. RALLOC: Stata module to design randomized controlled trials. EconPapers. 2011. http://EconPapers.repec.org/RePEc:boc:bocode:s319901.

[16] Bligh EG, Dyer WJ (1959). A rapid method of total lipid extraction and purification. Can J Biochem Physiol.

[17] Browning LM, Walker CG, Mander AP, West AL, Madden J, Gambell JM (2012). Incorporation of eicosapentaenoic and docosahexaenoic acids into lipid pools when given as supplements providing doses equivalent to typical intakes of oily fish. Am J Clin Nutr.

[18] Sewell GW, Hannun YA, Han X, Koster G, Bielawski J, Goss V (2012). Lipidomic profiling in Crohn’s disease: abnormalities in phosphatidylinositols, with preservation of ceramide, phosphatidylcholine and phosphatidylserine composition. Int J Biochem Cell Biol.

[19] Postle AD, Henderson NG, Koster G, Clark HW, Hunt AN (2011). Analysis of lung surfactant phosphatidylcholine metabolism in transgenic mice using stable isotopes. Chem Phys Lipids.

[20] Dondorp AM, Angus BJ, Chotivanich K, Silamut K, Ruangveerayuth R, Hardeman MR (1999). Red blood cell deformability as a predictor of anemia in severe falciparum malaria. Am J Trop Med Hyg.

[21] World Health Organisation. The WHO Child Growth Standards. 2006. http://www.who.int/childgrowth/en/.

[22] Maitland K, Berkley JA, Shebbe M, Peshu N, English M, Newton CR (2006). Children with severe malnutrition: can those at highest risk of death be identified with the WHO protocol?. PLoS Med.

[23] Moisi JC, Gatakaa H, Berkley JA, Maitland K, Mturi N, Newton CR (2011). Excess child mortality after discharge from hospital in Kilifi, Kenya: a retrospective cohort analysis. Bull World Health Organ.

[24] Kerac M, Bunn J, Chagaluka G, Bahwere P, Tomkins A, Collins S (2014). Follow-up of post-discharge growth and mortality after treatment for severe acute malnutrition (FuSAM study): a prospective cohort study. PLoS One.

[25] Chisti MJ, Graham SM, Duke T, Ahmed T, Faruque AS, Ashraf H (2014). Post-discharge mortality in children with severe malnutrition and pneumonia in bangladesh. PLoS One.

[26] Koletzko B, Lien E, Agostoni C, Bohles H, Campoy C, Cetin I (2008). The roles of long-chain polyunsaturated fatty acids in pregnancy, lactation and infancy: review of current knowledge and consensus recommendations. J Perinat Med.

[27] Carlson SE (2009). Early determinants of development: a lipid perspective. Am J Clin Nutr.

[28] Grantham-McGregor S (1995). A review of studies of the effect of severe malnutrition on mental development. J Nutr.

[29] UNICEF. Ready-to-use therapeutic food for children with severe acute malnutrition. Position Paper. UNICEF, 2013. [http://www.unicef.org/policyanalysis/files/UNICEF-Position-Paper_Ready-To-Use-Therapeutic-Food_June2013.pdf]

[30] Makhoul Z, Kristal AR, Gulati R, Luick B, Bersamin A, Boyer B (2010). Associations of very high intakes of eicosapentaenoic and docosahexaenoic acids with biomarkers of chronic disease risk among Yup’ik Eskimos. Am J Clin Nutr.

[31] Glaser C, Lattka E, Rzehak P, Steer C, Koletzko B (2011). Genetic variation in polyunsaturated fatty acid metabolism and its potential relevance for human development and health. Matern Child Nutr.

[32] Brenna JT, Salem N, Sinclair AJ, Cunnane SC (2009). Alpha-Linolenic acid supplementation and conversion to n-3 long-chain polyunsaturated fatty acids in humans. Prostaglandins Leukot Essent Fatty Acids.

[33] Brenna JT, Carlson SE (2014). Docosahexaenoic acid and human brain development: Evidence that a dietary supply is needed for optimal development. J Hum Evol.

[34] Grindel A, Staps F, Kuhnt K (2013). Cheek cell fatty acids reflect n-3 PUFA in blood fractions during linseed oil supplementation: a controlled human intervention study. Lipids Health Dis.

[35] Gibson RA, Neumann MA, Lien EL, Boyd KA, Tu WC (2013). Docosahexaenoic acid synthesis from alpha-linolenic acid is inhibited by diets high in polyunsaturated fatty acids. Prostaglandins Leukot Essent Fatty Acids.

[36] Gregory MK, Gibson RA, Cook-Johnson RJ, Cleland LG, James MJ (2011). Elongase reactions as control points in long-chain polyunsaturated fatty acid synthesis. PLoS One.

[37] Carlson SE, Werkman SH, Peeples JM, Cooke RJ, Tolley EA (1993). Arachidonic acid status correlates with first year growth in preterm infants. Proc Natl Acad Sci U S A.

[38] Gibson RA, Muhlhausler B, Makrides M (2011). Conversion of linoleic acid and alpha-linolenic acid to long-chain polyunsaturated fatty acids (LCPUFAs), with a focus on pregnancy, lactation and the first 2 years of life. Matern Child Nutr.

[39] Clark KJ, Makrides M, Neumann MA, Gibson RA (1992). Determination of the optimal ratio of linoleic acid to alpha-linolenic acid in infant formulas. J Pediatr.

[40] Hsieh JC, Liu L, Zeilani M, Ickes S, Trehan I, Maleta K, et al. High oleic ready-to-use therapeutic food maintains docosahexaenoic acid status in severe malnutrition: a randomized, blinded trial. J Pediatr Gastroenterol Nutr. 2015 [epublished ahead of print]. 10.1097/MPG.0000000000000741 PMC4483140 25633498

[41] Taha AY, Cheon Y, Faurot KF, Macintosh B, Majchrzak-Hong SF, Mann JD (2014). Dietary omega-6 fatty acid lowering increases bioavailability of omega-3 polyunsaturated fatty acids in human plasma lipid pools. Prostaglandins Leukot Essent Fatty Acids.

[42] Liou YA, King DJ, Zibrik D, Innis SM (2007). Decreasing linoleic acid with constant alpha-linolenic acid in dietary fats increases (n-3) eicosapentaenoic acid in plasma phospholipids in healthy men. J Nutr.

[43] von Schacky C. Omega-3 fatty Acids in cardiovascular disease - An uphill battle. Prostaglandins Leukot Essent Fatty Acids. 2014. [In press] 10.1016/j.plefa.2014.05.004 24935800

[44] McNamara RK (2009). Evaluation of docosahexaenoic acid deficiency as a preventable risk factor for recurrent affective disorders: current status, future directions, and dietary recommendations. Prostaglandins Leukot Essent Fatty Acids.

[45] Luxwolda MF, Kuipers RS, Sango WS, Kwesigabo G, Dijck-Brouwer DA, Muskiet FA (2012). A maternal erythrocyte DHA content of approximately 6 g% is the DHA status at which intrauterine DHA biomagnifications turns into bioattenuation and postnatal infant DHA equilibrium is reached. Eur J Nutr.

[46] Houssaini FZ, Foulon T, Iraqi MR, Payen N, Groslambert P (1999). Lipids, lipoproteins, and fatty acids during infantile marasmus in the Fes area of Morocco. Biochem Pharmacol.

[47] Koletzko B, Abiodun PO, Laryea MD, Bremer HJ (1986). Fatty acid composition of plasma lipids in Nigerian children with protein-energy malnutrition. Eur J Pediatr.

[48] Smit EN, Dijkstra JM, Schnater TA, Seerat E, Muskiet FA, Boersma ER (1997). Effects of malnutrition on the erythrocyte fatty acid composition and plasma vitamin E levels of Pakistani children. Acta Paediatr.

[49] Leichsenring M, Sutterlin N, Less S, Baumann K, Anninos A, Becker K (1995). Polyunsaturated fatty acids in erythrocyte and plasma lipids of children with severe protein-energy malnutrition. Acta Paediatr.

[50] Wolff JA, Margolis S, Bujdoso-Wolff K, Matusick E, MacLean WC (1984). Plasma and red blood cell fatty acid composition in children with protein-calorie malnutrition. Pediatr Res.

[51] Holman RT, Johnson SB, Mercuri O, Itarte HJ, Rodrigo MA, De Tomas ME (1981). Essential fatty acid deficiency in malnourished children. Am J Clin Nutr.

[52] Vajreswari A, Narayanareddy K, Rao PS (1990). Fatty acid composition of erythrocyte membrane lipid obtained from children suffering from kwashiorkor and marasmus. Metab Clin Exp.

[53] Franco VH, Hotta JK, Jorge SM, dos Santos JE (1999). Plasma fatty acids in children with grade III protein-energy malnutrition in its different clinical forms: marasmus, marasmic kwashiorkor, and kwashiorkor. J Trop Pediatr.

[54] Lauritzen L, Carlson SE (2011). Maternal fatty acid status during pregnancy and lactation and relation to newborn and infant status. Matern Child Nutr.

[55] Brenna JT, Varamini B, Jensen RG, Diersen-Schade DA, Boettcher JA, Arterburn LM (2007). Docosahexaenoic and arachidonic acid concentrations in human breast milk worldwide. Am J Clin Nutr.

[56] Michaelsen KF, Dewey KG, Perez-Exposito AB, Nurhasan M, Lauritzen L, Roos N (2011). Food sources and intake of n-6 and n-3 fatty acids in low-income countries with emphasis on infants, young children (6–24 months), and pregnant and lactating women. Matern Child Nutr.

[57] Prentice AM, Paul AA (2000). Fat and energy needs of children in developing countries. Am J Clin Nutr.

[58] Smit EN, Oelen EA, Seerat E, Boersma ER, Muskiet FA (2000). Fish oil supplementation improves docosahexaenoic acid status of malnourished infants. Arch Dis Child.

[59] van der Merwe LF, Moore SE, Fulford AJ, Halliday KE, Drammeh S, Young S (2013). Long-chain PUFA supplementation in rural African infants: a randomized controlled trial of effects on gut integrity, growth, and cognitive development. Am J Clin Nutr.

[60] Jones KD, Hunten-Kirsch B, Laving AM, Munyi CW, Ngari M, Mikusa J (2014). Mesalazine in the initial management of severely acutely malnourished children with environmental enteric dysfunction: a pilot randomized controlled trial. BMC Med.

