# Supplementary material for: Ready-to-use therapeutic food with elevated n-3 polyunsaturated fatty acid content, with or without fish oil, to treat severe acute malnutrition: a randomized controlled trial
Source: BMC Med. 2015 Apr 23;13:93. doi: 10.1186/s12916-015-0315-6 (PMC4407555; doi:10.1186/s12916-015-0315-6)
Supplement: Additional file 1: — Supplementary Information. [file 12916_2015_315_MOESM1_ESM.pdf]

**Ready-to-use therapeutic food with balanced essential fatty acid profile, with or without fish oil, to treat severe acute malnutrition: a randomized controlled trial**

## Online Supplementary Materials

**Contents**


2 Supplementary laboratory methods

4 Figure S1: Impact of breastfeeding on DHA accretion

5 Figure S2: Desaturase and elongase activity

6 Figure S3: Correlation between plasma phosphatidylcholine and erythrocyte fatty acids

7 Figure S4: Hematology

8 Table S1: RUTF composition

9 Table S2: Erythrocyte fatty acid composition

10 Table S3: Plasma phosphatidylcholine fatty acid composition

11 Table S4: Plasma phosphatidylcholine/erythrocyte fatty acid correlation

12 Table S5: CD3+ phosphatidylcholine fatty acid composition

13 Table S6: CD3+ phosphatidylethanolamine fatty acid composition

14 Table S7: Inflammatory indices

15 Table S8: T cell functional indices

## Supplementary laboratory methods

### T cell activation

T cell activation phenotypes (CD3+ CD4/8+ CD38+ HLA-DR+ lymphocytes with/without PD-1 expression) were evaluated by flow cytometric analysis of fresh whole blood. Blood was collected into sodium heparin vacutainers (BD) and processed within 2 hours of blood draw. Whole blood was stained at room temperature for 20 minutes in the dark. Staining antibodies were anti-CD3 V450, anti-CD4 PerCP-Cy5.5, anti-CD8 APC, anti-PD-1 PECy7 (all BD), anti-CD38 PE, anti-HLA-DR FITC (both Biolegend), and isotype controls (PE and PECy7 both against IgG1-kappa light chain from ebiosciences and Biolegend, respectively. Blood was lysed by addition of BD FACS Lysing buffer (1X) for 10 minutes, and washed three times in ice-cold PBS. Cells were fixed using BD Cellfix and read on a Beckman Coulter CyAn ADP the same day. BD CompBeads were used for compensation. Analysis was done in FlowJo. Gating strategy was as shown below:

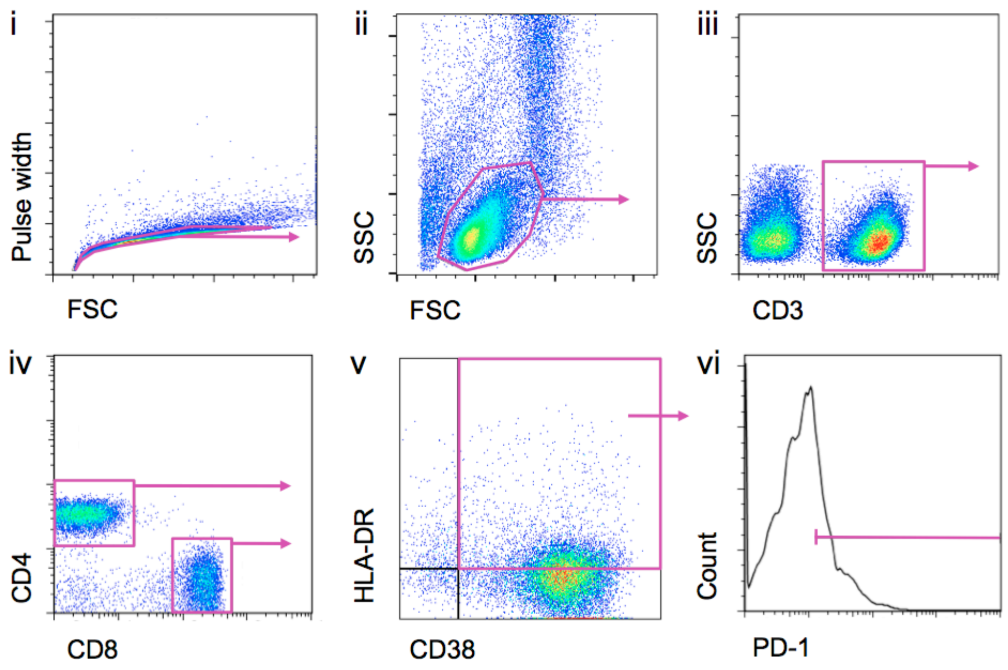


### T cell function

Whole blood was diluted 1 in 5 with RPMI (supplemented but serum-free) and stimulated in duplicate with phytohaemagglutinin (PHA, 5μg/ml, Sigma) or tetanus

toxoid (TT, 5Lf/ml, Serum Statens Institut) with or without IL-12 at 50 pg/ml. After 72 hours incubation at 37°C and 5% $CO_2$, supernatants were removed and interferon gamma (IFN-$\gamma$) concentration was assessed by ELISA (ebioscience) according to manufacturer's instructions.

**Inflammatory mediators in plasma**

Plasma IgG EndoCAb (Hycult Biotech) and serum insulin-like growth factor-1 (R&D Systems) were processed according to manufacturers' instructions. Muliplex ELISA (Luminex MAGPIX system) was performed against the following targets in heparinised plasma: Eotaxin (chemokine (C-C motif) ligand (CCL)-11), GRO$\alpha$ (growth regulated oncogene-$\alpha$, chemokine (C-X-C motif) ligand (CXCL)-1), Interferon-$\alpha$, Interleukin (IL)-1$\alpha$, IL-1 receptor antagonist (IL-1RA), IL-7, IL-8, IL-10, IL-15, IL-17a, IL-22, IL-31, IP-10 (interferon-$\gamma$ induced protein-10, CXCL10), MCP-1 (monocyte chemotactic protein-1, CCL2), MIP-1$\alpha$ (macrophage inflammatory protein-1$\alpha$, CCL3), MIP-1$\beta$ (CCL4), SDF-1 (stromal cell-derived factor-1, CXCL12), tumour necrosis factor-$\beta$ (TNF$\beta$) (Ebioscience). Soluble CD14 (sCD14) was measured using an in-house ELISA (capture clone 55-3, detection clone 3-C39 both from BD, recombinant standard from Sigma).

**<u>Supplementary Figure 1</u>: Impact of breastfeeding on DHA accretion.**

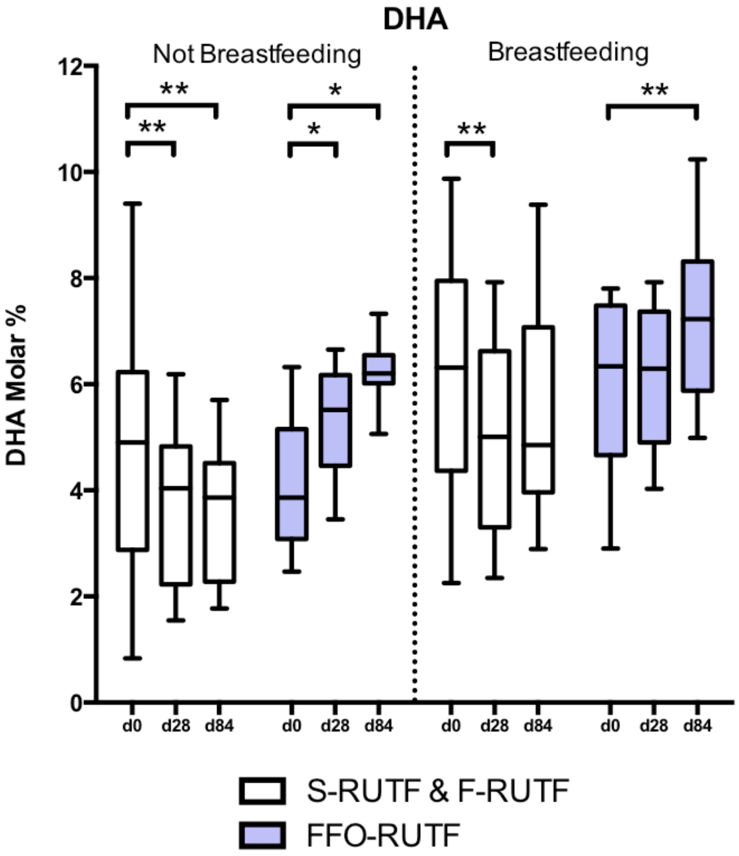


Erythrocyte DHA content during the trial is presented according to whether or not the participant was being breastfed at all on admission to the trial. Data are for the arm receiving fish oil (FFO-RUTF) or the other arms combined. * P≤0.05; ** P≤0.01; *** P≤0.001 (by sign test).

**<u>Supplementary Figure 2</u>: Desaturase and elongase activity.**

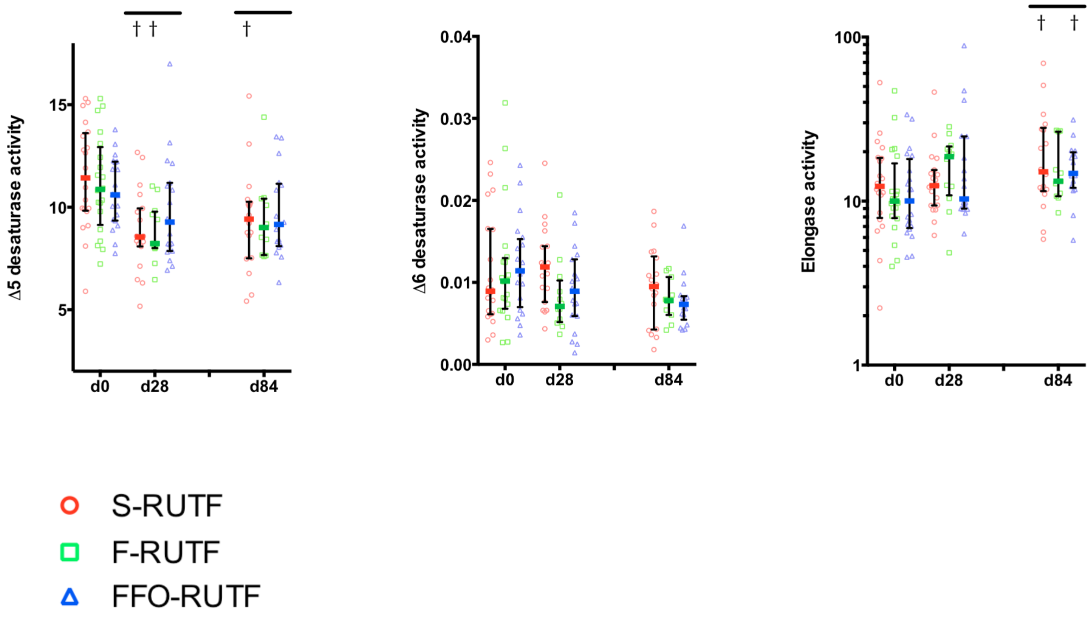


Calculated in erythrocyte lipids as n-6 product/substrate ratios as described in the text. For between-arm comparisons (ANOVA) at each time point, P>0.05 throughout. For within-arm comparison (sign test) to baseline values: † P≤0.05.

**<u>Supplementary Figure 3</u>: Correlation between plasma phosphatidylcholine and erythrocyte fatty acids.**

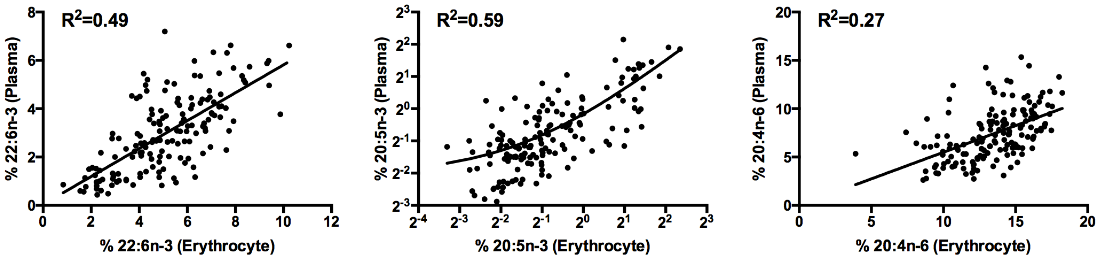


Data shown for DHA (22:6(n-3)), EPA (20:5(n-3)) and AA (20:4(n-6)).

**<u>Supplementary Figure 4</u>: Hematology.**

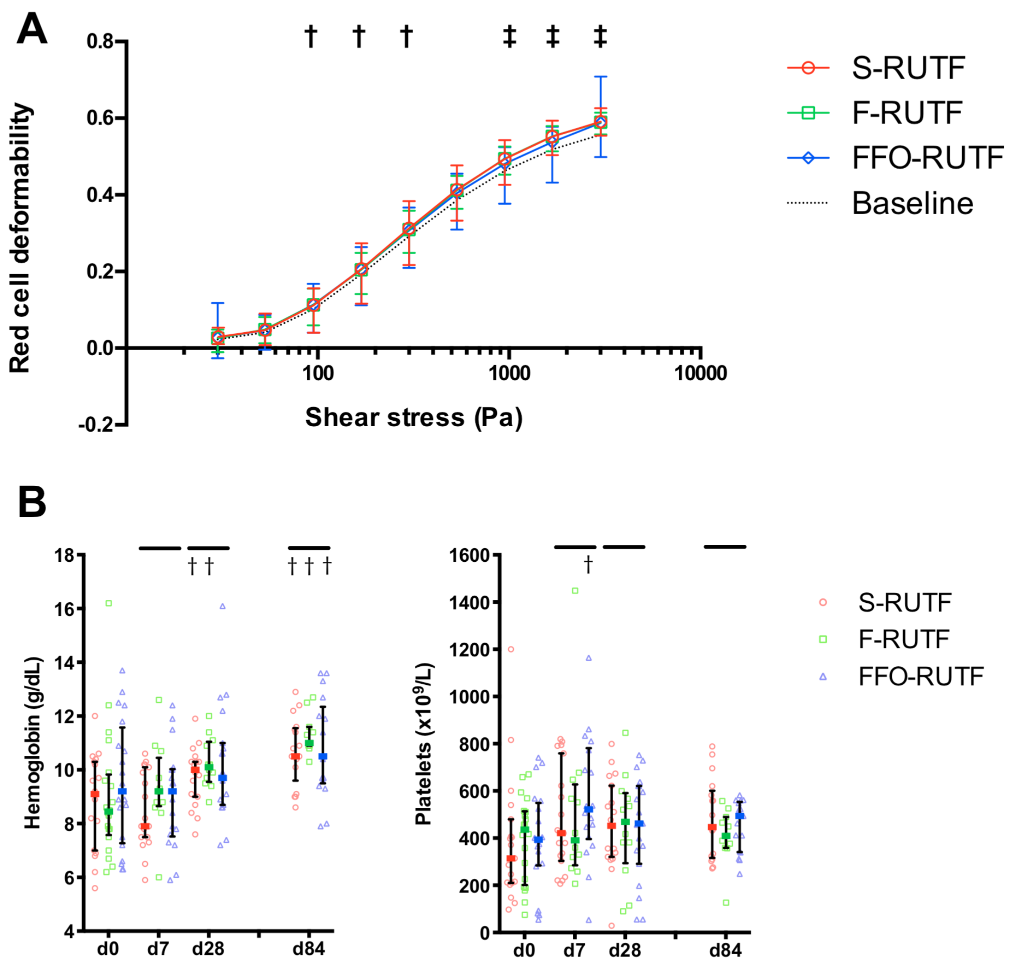


**A:** Red cell deformability measured by LORCA for the three arms at day 84, compared to the mean baseline values for all participants. † indicates P≤0.05 for the S-RUTF arm compared to baseline (sign test) and ‡ indicates P≤0.05 for the FFO-RUTF arm compared to baseline (sign test). All other tests (including between group by ANOVA) P>0.05. **B:** Hemoglobin and platelets by arm: For within-arm comparison (sign test) to baseline values: † P≤0.05.

**Supplementary Table 1: RUTF Composition.**

| | **S-RUTF** | **F-RUTF** | **FFO-RUTF** | ***Plumpy'nut*** | ***Fish oil*** |
|---|---|---|---|---|---|
| **14:0** | 0.69 | 0.00 | 0.28 | *0.75* | *7.94* |
| **15:0** | 0.05 | 0.04 | 0.04 | *0.05* | *0.00* |
| **16:0** | 30.78 | 30.54 | 30.17 | *28.82* | *18.58* |
| **16:1n-7** | 0.14 | 0.14 | 0.50 | *0.16* | *10.23* |
| **18:0** | 7.04 | 6.93 | 6.83 | *3.61* | *3.64* |
| **18:1n-9** | 43.39 | 38.55 | 37.65 | *48.57* | *11.30* |
| **t18:1n-9** | 0.00 | 0.78 | 0.76 | *1.03* | *4.86* |
| **18:2n-6** | 14.89 | 14.40 | 13.97 | *12.62* | *1.57* |
| **18:3n-6** | 0.08 | 0.13 | 0.14 | *0.06* | *0.29* |
| **18:3n-3** | 1.32 | 6.23 | 6.05 | *1.11* | *0.85* |
| **20:0** | 0.47 | 0.56 | 0.54 | *0.55* | *0.16* |
| **20:1n-9** | 0.34 | 0.45 | 0.54 | *1.03* | *3.00* |
| **20:2n-6** | 0.03 | 0.01 | 0.01 | *0.03* | *0.13* |
| **20:3n-6** | 0.00 | 0.00 | 0.00 | *0.00* | *0.12* |
| **20:4n-6** | 0.50 | 0.84 | 0.85 | *0.98* | *1.04* |
| **20:4n-3** | 0.00 | 0.05 | 0.13 | *0.09* | *2.22* |
| **20:5n-3** | 0.00 | 0.00 | 0.73 | *0.04* | *20.48* |
| **24:0** | 0.29 | 0.31 | 0.30 | *0.45* | *0.00* |
| **24:1n-9** | 0.00 | 0.02 | 0.02 | *0.06* | *0.00* |
| **22:5n-3** | 0.00 | 0.00 | 0.07 | *0.00* | *1.82* |
| **22:6n-3** | 0.00 | 0.00 | 0.42 | *0.00* | *11.78* |
| **n-6 total** | **15.50** | **15.38** | **14.97** | ***13.68*** | **3.14** |
| **n-3 total** | **1.32** | **6.28** | **7.40** | ***1.24*** | **37.14** |
| **n-6/n-3** | **11.72** | **2.45** | **2.02** | ***11.05*** | **0.08** |
| **n-6 %energy** | **8.21** | **8.15** | **7.93** | ***7.25*** | |
| **n-3 %energy** | **0.70** | **3.33** | **3.92** | ***0.66*** | |

Relative fatty acid content (% of total) of S-RUTF, F-RUTF and Plumpy'nut (for comparison) measured by gas chromatography. Encapsulated fish oil content was measured and the compositional equivalent of a single sachet of F-RUTF plus 2 fish oil capsules is calculated as FFO-RUTF (likely to be an overestimate since capsules were pierced and the oil squeezed out for consumption rather than being consumed whole). WHO/UNICEF recommendations are for n-6 as 3.0-10.0% total energy and n-3, 0.3-2.5% total energy.

**<u>Supplementary Table 2</u>: Erythrocyte fatty acid composition.**

| | Enrolment | | | Day 28 | | | Day 84 | | |
|---|---|---|---|---|---|---|---|---|---|
| | **S-RUTF** | **F-RUTF** | **FFO-RUTF** | **S-RUTF** | **F-RUTF** | **FFO-RUTF** | **S-RUTF** | **F-RUTF** | **FFO-RUTF** |
| **14:0** | 0.76 (0.47-1.00) * | 0.46 (0.36-0.61) * | 0.56 (0.33-0.88) * | 0.65 (0.26-1.13) | 0.36 (0.28-0.57) | 0.36 (0.30-0.58) | 0.56 (0.39-0.88) * | 0.33 (0.25-0.45) * | 0.39 (0.23-0.58) *b |
| **16:0** | 26.4 (25.3-27.5) | 26.5 (24.2-27.5) | 25.8 (24.7-26.7) | 25.1 (24.3-25.7) | 25.0 (24.1-26.2) | 24.8 (24.2-25.2) | 25.0 (24.5-25.7) a | 24.8 (23.4-25.5) c | 24.7 (24.2-25.1) |
| **16:1(n-7)** | 0.76 (0.50-1.15) | 0.60 (0.43-0.95) | 0.77 (0.36-0.96) | 0.43 (0.39-0.68) b | 0.38 (0.28-0.55) a | 0.39 (0.18-0.63) a | 0.39 (0.26-0.57) b | 0.36 (0.22-0.42) | 0.28 (0.20-0.38) c |
| **18:0** | 14.5 (12.5-16.1) ** | 13.0 (12.1-13.5) ** | 12.9 (12.2-13.6) ** | 14.9 (13.6-18.7) | 14.0 (13.6-15.0) | 13.4 (13.0-15.1) | 15.1 (13.7-17.5) ** | 14.3 (13.3-14.9) ** | 13.2 (12.6-13.7) ** |
| **18:1(n-9)** | 15.8 (14.5-17.0) | 16.2 (14.4-17.6) | 16.7 (14.6-17.6) | 15.9 (14.3-17.4) | 15.6 (14.8-16.2) | 15.0 (14.2-16.3) | 16.4 (13.9-16.9) | 15.4 (14.6-15.7) | 14.7 (13.6-15.9) b |
| **18:1(n-7)** | 1.35 (1.21-1.72) | 1.30 (1.10-1.61) | 1.35 (1.17-1.54) | 1.18 (1.05-1.37) b | 1.11 (1.03-1.18) b | 1.16 (0.98-1.28) c | 1.12 (1.04-1.20) b | 0.95 (0.87-1.21) c | 1.13 (0.95-1.21) c |
| **18:2(n-6)** | 10.3 (8.85-12.0) * | 12.0 (11.1-12.8) * | 11.6 (9.42-12.4) * | 10.4 (9.60-13.2) ** | 13.0 (12.1-13.9) ** | 11.5 (11.0-12.1) ** | 12.2 (10.6-13.2) * | 13.5 (13.0-14.3) * | 12.8 (11.5-13.6) *a |
| **18:3(n-6)** | 0.10 (0.06-0.14) | 0.12 (0.09-0.17) | 0.13 (0.08-0.16) | 0.11 (0.09-0.16) | 0.10 (0.07-0.13) | 0.11 (0.07-0.14) | 0.10 (0.06-0.14) | 0.11 (0.07-0.13) | 0.09 (0.08-0.11) |
| **18:3(n-3)** | 0.46 (0.26-0.73) | 0.58 (0.31-1.07) | 0.48 (0.26-1.05) | 0.55 (0.33-0.97) | 0.52 (0.42-0.64) | 0.55 (0.40-0.89) | 0.38 (0.22-0.51) | 0.55 (0.41-0.88) | 0.51 (0.26-0.62) |
| **20:0** | 0.91 (0.56-1.28) | 0.90 (0.35-1.57) | 0.98 (0.41-1.57) | 1.09 (0.65-1.84) | 1.01 (0.60-1.24) | 1.09 (0.46-1.65) | 0.86 (0.24-1.03) | 0.73 (0.44-1.07) | 0.74 (0.52-0.98) |
| **20:1(n-9)** | 0.18 (0.13-0.22) | 0.19 (0.14-0.29) | 0.40 (0.36-0.53) | 0.26 (0.19-0.36) b | 0.25 (0.18-0.40) | 0.30 (0.19-0.34) | 0.25 (0.16-0.40) | 0.29 (0.23-0.40) | 0.22 (0.16-0.27) |
| **20:2(n-6)** | 0.34 (0.25-0.41) ** | 0.40 (0.30-0.44) ** | 0.40 (0.36-0.53) ** | 0.39 (0.35-0.43) | 0.51 (0.41-0.51) a | 0.43 (0.38-0.49) | 0.38 (0.36-0.45) | 0.45 (0.39-0.46) | 0.38 (0.35-0.44) |
| **20:3(n-9)** | 0.08 (0.06-0.11) | 0.10 (0.06-0.13) | 0.09 (0.07-0.12) | 0.10 (0.07-0.12) | 0.09 (0.07-0.11) | 0.07 (0.04-0.10) | 0.09 (0.07-0.10) | 0.07 (0.07-0.11) | 0.08 (0.06-0.09) |
| **20:3(n-6)** | 1.29 (0.95-1.47) | 1.24 (1.02-1.47) | 1.34 (1.10-1.44) | 1.58 (1.25-1.86) *c | 1.48 (1.27-1.84) * | 1.28 (1.09-1.47) * | 1.61 (1.40-1.85) b | 1.57 (1.36-1.76) | 1.36 (1.15-1.60) |
| **20:4(n-6)** | 14.3 (11.7-14.6) | 13.9 (10.7-16.0) | 13.6 (12.9-15.3) | 14.4 (12.1-15.5) | 13.0 (11.1-14.5) | 12.3 (10.5-13.3) | 15.8 (13.7-16.4) *a | 13.9 (13.2-14.7) * | 13.2 (11.4-14.2) * |
| **20:4(n-3)** | 0.09 (0.06-0.11) | 0.07 (0.05-0.11) | 0.10 (0.05-0.16) | 0.08 (0.06-0.14) | 0.14 (0.11-0.17) c | 0.13 (0.07-0.15) | 0.08 (0.05-0.11) * | 0.12 (0.06-0.16) * | 0.09 (0.08-0.11) * |
| **20:5(n-3)** | 0.28 (0.22-0.48) | 0.37 (0.26-0.68) | 0.35 (0.28-0.50) | 0.32 (0.26-0.44) *** | 0.56 (0.49-0.74) *** | 2.08 (1.48-2.42) ***c | 0.37 (0.28-0.50) ***a | 0.73 (0.60-0.81) *** | 1.95 (1.54-2.63) ***c |
| **24:0** | 1.05 (0.80-1.31) | 1.01 (0.87-1.26) | 0.99 (0.87-1.13) | 1.03 (0.96-1.28) ** | 0.99 (0.76-1.11) **a | 0.82 (0.65-0.94) **b | 1.04 (0.88-1.14) *** | 0.70 (0.53-1.06) ***b | 0.60 (0.46-0.69) ***c |
| **24:1(n-9)** | 0.32 (0.24-0.37) * | 0.30 (0.26-0.37) * | 0.37 (0.32-0.43) * | 0.33 (0.30-0.39) | 0.39 (0.22-0.45) | 0.36 (0.28-0.44) | 0.39 (0.33-0.45) a | 0.35 (0.31-0.39) | 0.33 (0.27-0.44) |
| **22:5(n-3)** | 1.54 (1.24-1.90) | 1.86 (1.43-2.21) | 1.59 (1.42-2.01) | 1.70 (1.40-1.82) *** | 2.29 (1.99-2.80) *** | 3.07 (2.32-3.90) ***c | 1.66 (1.57-1.88) *** | 3.11 (2.53-3.44) ***c | 3.42 (3.03-3.91) ***c |
| **22:6(n-3)** | 5.37 (3.28-6.83) | 5.08 (3.98-6.39) | 5.41 (3.75-6.96) | 4.04 (2.23-5.90) **a | 4.55 (4.05-4.83) **a | 5.61 (4.90-6.75) **b | 3.88 (2.36-5.70) *** | 4.51 (3.92-4.85) *** | 6.30 (6.02-7.33) ***c |
| **Total n-6 + n-3** | 34.9 (30.9-36.6) | 35.5 (31.1-38.1) | 35.3 (31.8-38.1) | 34.6 (30.3-38.3) | 37.0 (34.3-38.5) | 39.0 (32.7-40.5) | 38.0 (31.2-39.9) **a | 38.8 (37.4-40.1) ** | 40.9 (40.1-41.7) **b |
| **Total LCn-6 + LCn-3** | 23.2 (19.0-26.0) | 23.3 (19.2-26.0) | 24.0 (20.2-25.7) | 22.7 (20.7-24.1) * | 22.1 (20.4-24.6) * | 25.9 (21.3-27.8) *a | 24.3 (20.3-27.2) ** | 23.9 (22.6-25.8) ** | 27.0 (25.9-28.6) **b |
| **n-6:n-3** | 3.24 (2.64-4.31) | 3.46 (2.70-3.92) | 3.21 (2.88-3.83) | 3.85 (2.90-5.21) ***a | 3.58 (3.06-3.90) *** | 2.15 (1.93-2.31) ***c | 4.48 (3.22-6.12) ***b | 3.23 (3.06-3.77) *** | 2.03 (1.90-2.23) ***c |
| **LCn-6:LCn-3** | 2.00 (1.75-2.90) | 1.96 (1.72-2.45) | 2.13 (1.80-2.41) | 2.44 (1.97-3.30) ***b | 2.05 (1.64-2.28) *** | 1.24 (1.11-1.41) ***c | 2.76 (1.96-3.80) ***b | 1.97 (1.77-2.23) *** | 1.20 (0.99-1.36) ***c |
| **Omega-3 Index** | 5.71 (3.48-7.27) | 5.41 (4.22-6.94) | 5.70 (4.47-7.42) | 4.41 (2.52-6.22) *** | 5.03 (4.76-5.59) *** | 7.92 (6.23-9.26) ***c | 4.23 (2.65-6.20) *** | 5.03 (4.73-5.56) *** | 9.18 (7.43-9.86) ***c |

Median (IQR). For between-arm comparisons (ANOVA) at each time point: * P≤0.05; ** P≤0.01; *** P≤0.001. For within-arm comparison (sign test) to baseline values: _a_ P≤0.05; _b_ P≤0.01; _c_ P≤0.001

**Supplementary Table 3: Plasma phosphatidylcholine fatty acid composition.**

| | Enrolment | | | Day 28 | | | Day 84 | | |
|---|---|---|---|---|---|---|---|---|---|
| | S-RUTF | F-RUTF | FFO-RUTF | S-RUTF | F-RUTF | FFO-RUTF | S-RUTF | F-RUTF | FFO-RUTF |
| **14:0** | 0.29 (0.20-0.40) | 0.30 (0.21-0.39) | 0.32 (0.23-0.46) | 0.22 (0.16-0.33) | 0.20 (0.16-0.28) | 0.30 (0.17-0.38) | 0.22 (0.12-0.32) a | 0.25 (0.17-0.39) | 0.24 (0.17-0.38) a |
| **16:0** | 33.7 (32.0-35.1) | 33.9 (31.9-35.2) | 33.4 (32.3-34.3) | 31.1 (29.7-31.9) a | 31.5 (30.4-32.9) a | 31.6 (30.6-32.9) | 30.8 (29.8-31.8) b | 31.9 (30.1-33.5) | 31.8 (30.7-32.3) b |
| **16:1(n-7)** | 0.38 (0.25-0.75) | 0.55 (0.33-0.90) | 0.59 (0.28-0.91) | 0.19 (0.17-0.32) b | 0.23 (0.17-0.31) | 0.21 (0.17-0.38) c | 0.26 (0.20-0.35) | 0.28 (0.16-0.45) | 0.21 (0.14-0.40) c |
| **18:0** | 12.8 (11.5-15.0) | 13.3 (11.6-14.0) | 13.4 (12.4-13.8) | 15.5 (14.0-16.0) a | 15.6 (13.7-16.4) b | 15.5 (14.6-16.5) c | 15.8 (14.1-16.4) a | 14.7 (13.4-17.3) | 15.4 (14.9-15.8) b |
| **18:1(n-9)** | 15.1 (11.9-18.5) | 15.7 (12.9-17.3) | 15.0 (11.8-18.8) | 11.7 (9.13-13.7) c | 11.0 (10.2-13.2) | 10.8 (9.76-12.3) c | 11.4 (9.10-11.7) *b | 11.5 (10.8-14.2) * | 10.7 (9.49-11.4) *c |
| **18:1(n-7)** | 1.92 (1.54-2.51) | 2.10 (1.68-2.20) | 1.68 (1.30-2.23) | 1.45 (1.24-1.71) | 1.39 (1.18-1.45) a | 1.45 (1.27-1.67) | 1.51 (1.38-1.69) a | 1.34 (1.13-1.83) | 1.47 (1.32-1.70) a |
| **18:2(n-6)** | 20.3 (17.7-22.4) | 21.8 (20.1-22.7) | 19.3 (21.4-24.4) | 24.7 (21.5-26.3) * | 25.8 (23.4-28.1) *c | 22.3 (21.4-25.2) * | 22.6 (19.8-25.2) | 23.0 (20.4-27.9) | 23.2 (21.0-24.1) |
| **18:3(n-6)** | 0.09 (0.05-0.15) | 0.09 (0.05-0.15) | 0.09 (0.07-0.16) | 0.10 (0.06-0.23) | 0.09 (0.06-0.13) | 0.08 (0.05-0.17) | 0.08 (0.04-0.10) | 0.06 (0.05-0.12) | 0.11 (0.05-0.14) |
| **18:3(n-3)** | 0.16 (0.09-0.26) | 0.17 (0.12-0.24) | 0.23 (0.11-0.36) | 0.20 (0.14-0.27) *** | 0.43 (0.30-0.57) *** | 0.57 (0.29-0.67) ***b | 0.11 (0.09-0.17) *** | 0.33 (0.24-0.62) *** | 0.23 (0.19-0.50) *** |
| **20:0** | 0.11 (0.08-0.14) | 0.11 (0.10-0.20) | 0.17 (0.07-0.29) | 0.18 (0.11-0.28) a | 0.15 (0.10-0.19) | 0.18 (0.12-0.26 | 0.12 (0.09-0.15) | 0.14 (0.11-0.29) | 0.14 (0.11-0.27) |
| **20:1(n-9)** | 0.19 (0.16-0.27) | 0.17 (0.12-0.21) | 0.18 (0.13-0.23) | 0.23 (0.18-0.30) | 0.23 (0.18-0.25) | 0.20 (0.16-0.27) | 0.19 (0.13-0.25) * | 0.24 (0.20-0.27) * | 0.20 (0.16-0.24) * |
| **20:2(n-6)** | 0.33 (0.24-0.39) | 0.29 (0.23-0.34) | 0.28 (0.22-0.35) | 0.32 (0.27-0.38) | 0.26 (0.21-0.35) | 0.31 (0.22-0.36) | 0.39 (0.30-0.45) | 0.30 (0.24-0.34) | 0.36 (0.28-0.45) a |
| **20:3(n-9)** | 0.13 (0.09-0.22) | 0.12 (0.07-0.21) | 0.11 (0.05-0.23) | 0.15 (0.07-0.17) | 0.08 (0.05-0.15) | 0.08 (0.05-0.16) | 0.11 (0.09-0.20) | 0.09 (0.05-0.20) | 0.09 (0.06-0.15) |
| **20:3(n-6)** | 1.72 (1.35-2.07) | 1.48 (1.25-1.96) | 1.73 (1.37-2.11) | 2.71 (2.00-3.17) **b | 1.88 (1.64-2.32) ** | 1.65 (1.11-2.12) ** | 2.41 (2.06-3.32) ***b | 1.71 (1.46-2.02) *** | 1.90 (1.43-2.01) *** |
| **20:4(n-6)** | 6.34 (5.53-8.36) | 6.87 (5.31-8.01) | 6.23 (4.77-9.04) | 7.71 (6.17-8.94) * | 5.10 (4.42-7.71) * | 5.70 (4.25-8.33) * | 9.73 (8.52-11.2) **a | 6.87 (5.75-9.38) ** | 7.82 (6.10-9.84) ** |
| **20:4(n-3)** | 0.09 (0.06-0.13) | 0.07 (0.04-0.12) | 0.08 (0.04-0.14) | 0.09 (0.06-0.14) | 0.12 (0.09-0.18) b | 0.12 (0.08-0.16) | 0.07 (0.05-0.09) * | 0.08 (0.07-0.13) *b | 0.12 (0.07-0.13) *a |
| **20:5(n-3)** | 0.37 (0.22-0.60) | 0.44 (0.29-0.61) | 0.37 (0.29-0.49) | 0.44 (0.35-0.57) *** | 0.69 (0.39-0.84) *** | 1.74 (0.71-2.40) ***c | 0.44 (0.32-0.52) *** | 0.88 (0.51-0.94) *** | 1.01 (0.68-1.96) ***c |
| **24:0** | 0.27 (0.18-0.40) | 0.23 (0.17-0.29) | 0.25 (0.16-0.34) | 0.33 (0.21-0.44) *** | 0.21 (0.14-0.30) *** | 0.14 (0.11-0.22) ***a | 0.40 (0.36-0.52) *** | 0.25 (0.19-0.33) *** | 0.18 (0.15-0.22) ***a |
| **24:1(n-9)** | 0.12 (0.07-0.16) | 0.13 (0.08-0.17) | 0.12 (0.09-0.18) | 0.10 (0.08-0.13) | 0.09 (0.08-0.17) | 0.10 (0.07-0.15) | 0.11 (0.87-0.14) | 0.13 (0.10-0.17) | 0.13 (0.09-0.17) |
| **22:5(n-3)** | 0.69 (0.39-1.29) | 0.71 (0.53-0.90) | 0.71 (0.51-0.82) | 0.76 (0.53-1.00) *** | 1.02 (0.69-1.19) ***a | 1.64 (0.94-1.92) ***b | 0.91 (0.66-1.11) * | 1.05 (0.83-1.33) * | 1.32 (1.09-1.49) *b |
| **22:6(n-3)** | 2.51 (1.00-4.63) | 2.63 (1.11-3.66) | 2.35 (1.15-3.69) | 2.23 (1.02-3.35) *** | 2.08 (1.38-2.97) *** | 4.26 (3.27-4.98) ***c | 2.36 (1.52-4.43) ** | 2.70 (2.05-2.96) ** | 4.15 (3.57-5.36) ** |
| **Total n-6+n-3** | 33.6 (30.3-37.1) | 34.7 (31.3-36.2) | 35.3 (31.4-38.2) | 39.1 (37.1-41.9) c | 39.9 (36.7-41.2) a | 38.9 (38.0-40.4) b | 40.5 (38.9-41.6) c | 39.6 (36.2-41.3) | 40.4 (39.3-42.9) b |
| **Total LCn-6+LCn-3** | 12.3 (9.45-15.6) | 13.3 (9.35-14.8) | 11.7 (9.77-16.5) | 13.5 (12.5-16.3) * | 11.6 (9.40-15.3) * | 16.2 (13.2-17.9) *a | 16.9 (15.2-19.9) a | 13.3 (12.9-15.6) | 16.3 (15.2-20.6) b |
| **n-6:n-3** | 8.04 (4.17-13.3) | 7.72 (5.27-11.0) | 7.93 (6.67-11.1) | 8.24 (7.15-14.7) *** | 7.82 (6.69-8.98) *** | 3.60 (3.29-4.86) ***c | 9.20 (5.52-12.4) *** | 6.63 (6.17-7.57) *** | 4.40 (3.86-5.64) ***b |
| **LCn-6:LCn-3** | 2.73 (1.67-3.72) | 2.59 (1.71-3.48) | 2.41 (2.15-3.08) | 2.93 (2.08-4.64) *** | 1.94 (1.74-2.32) *** | 1.01 (0.73-1.43) ***c | 3.41 (2.48-4.68) *** | 2.25 (1.72-2.29) *** | 1.50 (1.05-1.75) ***b |

Median (IQR). For between-arm comparisons (ANOVA) at each time point: * P≤0.05; ** P≤0.01; *** P≤0.001. For within-arm comparison (sign test) to baseline values: *a* P≤0.05; *b* P≤0.01; *c* P≤0.001

**<u>Supplementary Table 4</u>: Plasma phosphatidylcholine/erythrocyte fatty acid correlation.**

| | Enrolment | Day 28 | Day 84 | Total |
|---|---|---|---|---|
| **14:0** | 0.07 | 0.10 | 0.08 | 0.09 |
| **16:0** | 0.00 | 0.01 | 0.00 | 0.04 |
| **16:1(n-7)** | 0.21 | 0.03 | 0.12 | 0.26 |
| **18:0** | 0.13 | 0.01 | 0.08 | 0.07 |
| **18:1(n-9)** | 0.39 | 0.35 | 0.27 | 0.32 |
| **18:1(n-7)** | 0.07 | 0.22 | 0.29 | 0.22 |
| **18:2(n-6)** | 0.21 | 0.09 | 0.27 | 0.19 |
| **18:3(n-6)** | 0.01 | 0.00 | 0.01 | 0.00 |
| **18:3(n-3)** | 0.00 | 0.00 | 0.13 | 0.01 |
| **20:0** | 0.03 | 0.01 | 0.02 | 0.01 |
| **20:1(n-9)** | 0.05 | 0.06 | 0.07 | 0.01 |
| **20:2(n-6)** | 0.10 | 0.09 | 0.10 | 0.08 |
| **20:3(n-9)** | 0.00 | 0.02 | 0.01 | 0.00 |
| **20:3(n-6)** | 0.25 | 0.21 | 0.47 | 0.34 |
| **20:4(n-6)** | 0.22 | 0.28 | 0.29 | 0.27 |
| **20:4(n-3)** | 0.01 | 0.25 | 0.00 | 0.05 |
| **20:5(n-3)** | 0.08 | 0.71 | 0.54 | 0.59 |
| **24:0** | 0.10 | 0.17 | 0.45 | 0.16 |
| **24:1(n-9)** | 0.00 | 0.01 | 0.00 | 0.00 |
| **22:5(n-3)** | 0.02 | 0.26 | 0.29 | 0.25 |
| **22:6(n-3)** | 0.52 | 0.46 | 0.55 | 0.49 |
| **Total n-6 + n-3** | 0.12 | 0.03 | 0.02 | 0.11 |
| **Total LCn-6 + LCn-3** | 0.29 | 0.19 | 0.12 | 0.25 |
| **n-6:n-3** | 0.50 | 0.86 | 0.71 | 0.62 |
| **LCn-6:LCn-3** | 0.56 | 0.87 | 0.83 | 0.74 |

Data are R-squared values from linear regression analyses between erythrocyte GC data and plasma phosphatidylcholine GC data for each molecular species at the three time points and (total) for all the values at the three timepoints combined.

**<u>Supplementary Table 5</u>: CD3+ phosphatidylcholine fatty acid composition.**

| | Enrolment | | | Day 84 | | |
|---|---|---|---|---|---|---|
| | S-RUTF | F-RUTF | FFO-RUTF | S-RUTF | F-RUTF | FFO-RUTF |
| **16:0 & 14:0** | 0.50 (0.34-0.75) | 0.55 (0.44-0.69) | 0.61 (0.45-0.93) | 0.67 (0.56-1.10) | 0.51 (0.39-0.74) | 0.67 (0.45-1.03) |
| **16:0a & 16:0** | 0.70 (0.65-0.86) | 0.84 (0.56-1.08) | 0.75 (0.59-1.04) | 0.84 (0.65-1.07) | 0.84 (0.68-0.96) | 0.78 (0.61-0.87) |
| **16:0 & 16:1** | 1.10 (0.90-1.27) | 1.17 (1.00-1.44) | 1.10 (0.93-1.32) | 1.10 (0.90-1.46) | 0.93 (0.82-1.15) *a* | 0.93 (0.79-1.12) |
| **16:0 & 16:0** | 5.39 (5.36-8.99) | 8.95 (6.06-9.44) | 7.09 (6.43-7.54) | 8.82 (6.24-9.95) | 7.55 (6.32-9.98) | 8.61 (6.68-10.1) |
| **16:0a & 18:1** | 1.50 (1.27-1.72) | 1.62 (1.19-1.78) | 1.31 (1.17-1.72) | 1.51 (1.20-1.62) | 1.57 (1.13-1.68) | 1.32 (1.19-1.59) |
| **16:0 & 18:2** | 7.46 (6.75-8.67) | 7.45 (6.29-8.94) | 7.71 (6.76-8.60) | 8.25 (6.08-9.24) | 8.34 (7.87-9.41) | 8.48 (7.10-9.82) |
| **16:0 & 18:1** | 14.8 (14.2-16.9) | 16.4 (14.5-17.1) | 15.5 (13.2-17.2) | 12.7 (11.7-14.5) | 14.3 (13.7-15.2) | 13.6 (12.3-14.9) |
| **18:0 & 16:0** | 0.64 (0.51-0.84) | 0.86 (0.58-1.04) | 0.76 (0.46-1.02) | 0.78 (0.71-0.93) | 0.78 (0.63-0.91) | 0.88 (0.80-1.05) |
| **16:0a & 20:4** | 0.86 (0.79-1.16) | 0.83 (0.72-0.91) | 0.85 (0.75-1.15) | 1.05 (0.86-1.12) ** | 0.76 (0.65-0.86) ** | 0.63 (0.60-0.97) ** |
| **16:0 & 20:5** | 0.21 (0.17-0.31) | 0.26 (0.21-0.36) | 0.24 (0.18-0.30) | 0.29 (0.25-0.32) ** | 0.39 (0.27-0.42) ** | 0.44 (0.33-1.00) ***b* |
| **16:0 & 20:4** | 8.03 (7.24-9.45) | 7.49 (6.27-10.1) | 6.81 (5.71-7.92) | 9.77 (8.75-10.4) ** | 7.84 (6.81-8.74) ** | 7.11 (6.13-8.53) ** |
| **18:1 & 18:2** | 5.93 (5.44-6.67) | 5.63 (4.97-6.74) | 5.14 (3.61-6.25) | 4.99 (3.82-6.11) | 6.34 (5.16-7.89) | 5.79 (4.97-6.44) |
| **18:0 & 18:2** | 7.69 (6.80-8.52) | 8.09 (7.57-8.54) | 6.90 (5.99-8.06) | 8.03 (6.08-8.97) | 8.77 (7.74-10.2) | 7.77 (7.74-9.52) *b* |
| **18:0 & 18:1** | 2.47 (1.86-2.95) | 3.01 (2.25-3.22) | 2.93 (1.94-3.72) | 2.23 (1.94-2.53) | 2.67 (2.45-2.90) | 2.41 (1.95-3.13) |
| **18:1 & 20:4** | 1.17 (1.00-1.47) | 1.08 (0.87-1.21) | 1.10 (0.99-1.25) | 1.27 (1.13-1.39) ** | 1.26 (1.06-1.45) ** | 0.97 (0.97-1.20) ** |
| **16:0 & 22:6** | 1.11 (0.89-1.36) | 1.09 (0.89-1.42) | 1.14 (1.00-1.42) | 1.25 (1.08-1.58) * | 1.09 (1.00-1.19) * | 1.44 (1.34-1.64) * |
| **18:1 & 20:4** | 4.25 (3.79-5.19) | 3.74 (3.54-4.23) | 3.59 (3.10-4.64) | 3.96 (3.70-4.62) | 3.64 (3.2504.61) | 3.67 (3.33-4.01) |
| **18:0 & 20:4** | 7.37 (6.79-8.26) * | 6.65 (5.37-7.61) * | 6.06 (5.15-7.25) * | 8.54 (6.89-10.2) * | 6.99 (6.13-8.66) * | 6.28 (4.93-7.68) * |
| **18:0 & 20:3** | 1.73 (1.34-1.98) | 1.54 (1.22-1.70) | 1.67 (1.26-1.95) | 1.60 (1.47-1.86) | 1.74 (1.67-2.09) | 1.80 (1.70-2.08) |
| **18:0 & 22:6** | 0.77 (0.64-0.97) | 0.72 (0.58-0.85) | 0.76 (0.70-0.89) | 0.79 (0.65-1.05) | 0.69 (0.62-0.80) | 0.76 (0.70-0.96) |
| **18:0 & 22:5** | 0.78 (0.70-1.10) | 0.76 (0.65-0.81) | 0.83 (0.72-0.98) | 0.80 (0.63-0.83) | 0.81 (0.65-0.96) | 0.70 (0.66-0.78) |
| | | | | | | |
| **20:4(n-6)** | 10.8 (9.76-12.8) * | 9.50 (8.71-11.4) * | 9.54 (8.17-11.0) * | 12.4 (11.1-13.5) ** | 10.4 (9.51-11.3) ** | 9.64 (8.03-10.5) ** |
| **20:5(n-3)** | 0.11 (0.09-0.16) | 0.13 (0.10-0.18) | 0.12 (0.09-0.15) | 0.15 (0.12-0.16) ** | 0.19 (0.13-0.21) ** | 0.22 (0.16-0.50) ***b* |
| **22:6(n-3)** | 0.87 (0.84-1.24) | 0.90 (0.79-1.08) | 0.94 (0.86-1.15) | 1.02 (0.89-1.30) | 0.91 (0.81-0.98) | 1.12 (1.02-1.30) |

Median (IQR). For between-arm comparisons (ANOVA) at each time point: * P≤0.05; ** P≤0.01; *** P≤0.001. For within-arm comparison (sign test) to baseline values: *a* P≤0.05; *b* P≤0.01; *c* P≤0.001. Output data are mass spectra corresponding to PC (or PE, below) diacylglycerol components. Predicted mass identity is listed and total percentage content of AA, EPA and DHA is listed.

**<u>Supplementary Table 6</u>: CD3+ phosphatidylethanolamine fatty acid composition.**

| | Enrolment | | | Day 28 | | |
|---|---|---|---|---|---|---|
| | S-RUTF | F-RUTF | FFO-RUTF | S-RUTF | F-RUTF | FFO-RUTF |
| **16:1 & 16:1** | 0.32 (0.15-0.37) | 0.22 (0.12-0.36) | 0.29 (0.23-0.42) | 0.24 (0.17-0.50) | 0.43 (0.21-0.72) | 0.23 (0.11-0.34) |
| **16:0 & 16:1** | 0.13 (0.10-0.20) | 0.19 (0.15-0.26) | 0.24 (0.16-0.37) | 0.14 (0.12-0.43) *a* | 0.20 (0.10-0.62) | 0.12 (0.10-0.17) |
| **16:0 & 16:0** | 1.31 (0.85-1.82) | 0.85 (0.38-1.06) | 1.39 (0.88-2.31) | 1.35 (1.00-2.29) | 0.86 (0.72-1.90) | 1.97 (1.11-3.21) |
| **16:1 & 18:2** | 0.16 (0.10-0.22) | 0.14 (0.11-0.20) | 0.21 (0.18-0.25) | 0.16 (0.09-0.37) | 0.18 (0.13-0.47) | 0.18 (0.10-0.24) |
| **16:0 & 18:2** | 1.66 (1.46-2.20) | 2.09 (1.69-2.45) | 1.80 (1.53-2.24) | 1.68 (1.28-2.41) | 1.94 (1.47-2.96) | 1.80 (1.53-2.01) |
| **16:0 & 18:1** | 2.47 (2.01-2.90) | 2.65 (2.29-3.19) | 2.61 (2.10-3.10) | 2.36 (1.71-2.80) | 2.75 (2.01-3.69) | 2.14 (1.92-2.48) |
| **16:0 & 20:4** | 4.51 (3.54-4.93) | 4.97 (4.53-5.60) | 4.86 (4.01-5.34) | 4.25 (3.34-4.70) | 3.98 (3.34-4.78) | 4.03 (3.68-4.67) |
| **18:1 & 18:2** | 2.33 (1.76-2.84) | 2.51 (1.74-3.80) | 2.59 (1.99-3.14) | 2.38 (1.49-2.86) | 2.93 (2.20-4.19) | 2.67 (2.44-2.84) |
| **18:1 & 18:1** | 6.42 (5.48-7.55) | 7.95 (6.05-9.11) | 6.85 (6.76-7.47) | 7.27 (5.96-7.81) | 8.87 (6.33-10.3) | 8.97 (6.77-9.39) |
| **18:0 & 18:1** | 2.86 (2.39-3.43) | 3.27 (3.06-3.62) | 3.29 (3.05-3.67) | 2.77 (1.90-3.03) | 2.95 (2.46-3.78) | 3.10 (2.27-3.91) |
| **18:0p & 20:4** | 1.05 (0.97-1.08) | 0.95 (0.81-1.08) | 0.92 (0.83-1.18) | 0.85 (0.71-1.24) | 0.87 (0.83-1.00) | 0.91 (0.78-1.08) |
| **18:0p & 20:3** | 0.88 (0.74-1.01) | 0.80 (0.68-0.98) | 0.93 (0.87-1.13) | 0.81 (0.66-0.93) | 0.72 (0.62-0.87) | 0.88 (0.60-0.96) |
| **16:0 & 22:6** | 1.93 (1.31-2.20) | 2.24 (1.78-2.75) | 2.11 (1.67-2.73) | 2.05 (1.82-2.62) | 1.77 (1.43-2.11) | 2.28 (1.98-2.99) |
| **18:1 & 20:4** | 5.12 (4.21-5.73) | 5.15 (4.42-5.53) | 4.96 (4.50-5.23) | 4.27 (3.05-4.80) | 4.08 (3.38-4.93) | 4.86 (3.92-5.70) |
| **18:0 & 20:4** | 27.5 (22.2-32.6) | 29.1 (27.2-29.5) | 26.0 (22.3-28.4) | 26.4 (21.2-33.4) | 24.9 (21.7-29.6) | 25.5 (20.9-28.5) |
| **18:0 & 20:3** | 3.12 (2.76-3.90) | 3.50 (2.97-3.94) | 3.23 (2.57-3.83) | 3.01 (2.48-3.38) | 3.16 (2.45-3.40) | 3.11 (2.68-3.62) |
| **18:1 & 22:6** | 2.26 (1.26-2.60) | 2.11 (1.47-2.64) | 1.88 (1.67-2.78) | 1.95 (1.68-2.60) | 2.02 (1.64-2.14) | 2.00 (1.63-2.36) |
| **18:0 & 22:6** | 3.30 (2.46-4.06) | 3.37 (2,87-4.62) | 3.35 (2.64-4.20) | 3.59 (3.05-4.48) | 2.70 (2.42-2.90) | 3.59 (2.71-4.97) |
| **18:0 & 22:5** | 2.75 (2.39-3.18) | 3.12 (2.45-3.29) | 2.35 (2.11-3.04) | 2.74 (2.04-3.01) | 2.50 (2.45-3.27) | 3.04 (2.64-3.79) |
| **18:0 & 22:4** | 2.57 (2.20-3.36) * | 2.46 (2.20-2.66) * | 2.01 (1.66-2.47) * | 2.65 (1.78-2.95) | 2.19 (1.64-2.34) | 1.65 (1.52-1.98) |
| | | | | | | |
| **20:4(n-6)** | 19.3 (16.0-22.1) | 20.4 (20.0-21.1) | 18.8 (16.1-19.4) | 18.2 (13.8-21.3) | 17.5 (14.5-20.3) | 17.4 (15.0-19.3) |
| **22:6(n-3)** | 3.64 (2.51-4.52) | 3.71 (3.30-5.06) | 3.46 (3.23-4.57) | 3.81 (3.31-4.90) | 3.12 (2.81-3.43) | 3.75 (3.17-5.21) |

Median (IQR). For between-arm comparisons (ANOVA) at each time point: * P≤0.05; ** P≤0.01; *** P≤0.001. For within-arm comparison (sign test) to baseline values: *a* P≤0.05; *b* P≤0.01; *c* P≤0.001. Output data are mass spectra corresponding to PE (or PC, above) diacylglycerol components. Predicted mass identity is listed and total percentage content of AA and DHA is listed.

## <u>Supplementary Table 7</u>: Inflammatory indices.

| | Enrolment | | | Day 7 | | | Day 28 | | | Day 84 | | |
|---|---|---|---|---|---|---|---|---|---|---|---|---|
| | S-RUTF | F-RUTF | FFO-RUTF | S-RUTF | F-RUTF | FFO-RUTF | S-RUTF | F-RUTF | FFO-RUTF | S-RUTF | F-RUTF | FFO-RUTF |
| **IGF-1** | 44.5 (30.7-58.2) | 50.3 (30.2-69.8) | 44.7 (15.5-62.7) | | | | 140 (63.1-169) *c* | 143 (77.9-275) *c* | 114 (45.3-143) *b* | 103 (65.9-180) *b* | 94.4 (74.2-138) | 68.8 (47.9-114) *a* |
| **Platelets** | 315 (210-480) | 437 (210-513) | 394 (287-541) | 421 (304-759) | 391 (297-608) | 522 (405-772) *a* | 452 (321-622) | 470 (323-583) | 462 (291-621) | 447 (323-582) | 410 (359-490) | 494 (344-548) |
| **WCC** | 10.6 (8.50-14.8) | 10.7 (8.65-14.0) | 10.7 (9.40-15.9) | 13.2 (8.80-15.9) | 11.9 (9.90-13.5) | 12.7 (10.6-14.5) | 12.0 (5.90-16.3) | 13.3 (8.85-15.5) | 11.5 (8.90-14.0) | 11.9 (9.90-14.1) | 12.5 (9.10-14.5) | 11.1 (8.20-13.8) |
| **Neutrophils** | 3.25 (1.78-4.30) | 3.24 (1.64-4.18) | 3.71 (2.94-4.28) | 4.85 (2.73-7.60) | 3.62 (2.71-4.93) | 4.47 (4.00-5.10) | 3.20 (2.45-5.61) | 3.97 (3.63-5.55) | 3.87 (3.08-4.61) | 3.22 (2.39-4.41) | 2.94 (2.66-5.35) | 3.45 (2.36-4.37) |
| **Lymphocytes** | 5.93 (4.24-6.91) | 6.74 (5.36-8.35) | 5.84 (4.84-7.66) | 4.80 (3.34-7.91) | 5.60 (5.24-8.53) | 5.92 (5.05-8.36) | 5.39 (3.53-8.54) | 6.82 (4.55-9.23) | 6.47 (3.86-7.29) | 6.81 (4.23-8.30) | 8.01 (5.17-8.36) | 6.48 (4.85-7.70) |
| **Hb** | 9.10 (7.00-10.3) | 8.45 (7.65-9.75) | 9.20 (7.35-11.4) | 7.90 (7.50-10.1) | 9.20 (8.70-10.2) | 9.20 (7.60-10.0) | 10.0 (9.00-10.3) *b* | 10.1 (9.60-11.0) *a* | 9.70 (8.70-11.0) | 10.5 (10.1-11.5) *b* | 11.0 (10.9-11.6) *a* | 10.5 (9.60-12.0) *a* |
| **EndoCAb** | 39.7 (21.3-85.2) | 62.3 (33.4-138) | 60.2 (38.2-103) | | | | 51.0 (27.6-80.0) | 70.7 (29.4-121) | 61.8 (37.6-123) | 39.9 (17.0-78.5) | 60.3 (44.3-141) | 70.1 (29.0-115) |
| **sCD14** | 2369 (1862-2784) | 2444 (2240-3399) | 2298 (1965-2897) | | | | 2870 (2532-3827) *b* | 3113 (2583-3351) | 2551 (2246-3053) | 2861 (2499-3188) *a* | 2625 (2154-3277) | 2567 (2036-3206) |
| **MCP-1** | 16.9 (12.3-20.1) | 25.4 (12.3-63.8) | 18.3 (9.25-23.9) | | | | 10.7 (6.23-19.0) | 17.1 (9.16-30.7_) | 10.7 (9.22-20.0) | 13.2 (9.58-21.2) | 15.6 (6.96-24.8) | 10.2 (7.35-17.8) |
| **SDF-1** | 265 (199-314) | 245 (193-295) | 237 (200-307) | | | | 230 (192-258) | 223 (137-265) | 213 (165-253) | 108 (151-257) *a* | 222 (136-268) | 203 (174-238) *b* |
| **CXCL10** | 68.0 (36.7-93.5) | 45.4 (25.9-98.8) | 32.7 (19.4-58.3) | | | | 42.1 (25.6-77.9) | 44.0 (39.1-57.1) | 28.6 (14.3-42.3) | 39.1 (24.5-57.8) *b* | 31.7 (18.6-41.3) *a* | 38.5 (31.4-54.3) |
| **IL-7** | 2.47 (1.65-3.26) | 2.52 (1.59-4.65) | 2.62 (1.51-3.34) | | | | 3.89 (3.05-5.21) *a* | 4.30 (2.67-4.89) | 3.04 (2.04-5.39) | 1.42 (1.42-1.42) | 1.42 (1.42-1.42) | 1.42 (1.42-1.42) |
| **IL-8** | 16.7 (9.07-26.9) | 15.2 (11.4-25.6) | 12.5 (8.64-15.7) | | | | 9.91 (6.60-14.1) | 7.18 (6.44-11.5) | 6.44 (9.10-15.2) | 6.44 (6.44-6.74) *b* | 6.44 (6.44-6.77) | 6.44 (6.44-11.5) |
| **IL-10** | 4.31 (2.43-8.18) | 4.65 (2.64-5.85) | 3.09 (2.14-7.27) | | | | 2.48 (2.14-6.91) | 2.83 (2.14-5.44) | 3.22 (2.14-5.66) | 2.14 (2.14-3.54) *b* | 2.23 (2.14-4.19) *a* | 2.14 (2.14-2.20) *a* |
| **Eotaxin** | 10.2 (6.93-14.2) | 9.67 (8.51-11.8) | 10.6 (8.19-15.5) | | | | 9.38 (6.01-12.2) | 8.45 (6.34-10.0) | 7.90 (6.35-10.9) | 10.8 (7.47-13.5) | 8.38 (7.49-12.9) | 10.2 (8.56-12.4) |
| **IL-17a** | 5.12 (5.12-5.12) | 5.12 (5.12-5.12) | 5.12 (5.12-5.12) | | | | 5.12 (5.12-6.14) | 5.12 (5.12-5.12) | 5.12 (5.12-7.06) | 5.12 (5.12-5.12) | 5.12 (5.12-5.12) | 5.12 (5.12-5.12) |
| **IL-31** | 34.1 (34.1-34.1) | 34.1 (34.1-34.1) | 34.1 (34.1-34.1) | | | | 34.1 (34.1-34.1) | 34.1 (34.1-34.1) | 34.1 (34.1-34.1) | 34.1 (34.1-34.1) | 34.1 (34.1-34.1) | 34.1 (34.1-34.1) |
| **IL-1RA** | 1054 (2549-6182) | 2322 (972-4289) | 1228 (674-1882) | | | | 1708 (864-4446) * | 1307 (997-1989) * | 1036 (511-1665) * | 1600 (743-2584) | 1246 (622-2150) | 1086 (572-1946) |
| **MIP-1b** | 195 (94.3-287) | 169 (93.6-300) | 116 (94.9-209) | | | | 119 (78.8-215) | 112 (81.5-166) | 120 (94.5-205) | 140 (111-181) | 101 (60.1-168) | 120 (96.9-147) |
| **IFN-a** | 1.42 (1.42-1.42) | 1.42 (1.42-1.42) | 1.42 (1.42-1.42) | | | | 1.42 (1.42-1.42) | 1.42 (1.42-1.42) | 1.42 (1.42-1.42) | 1.42 (1.42-1.42) | 1.42 (1.42-1.42) | 1.42 (1.42-1.42) |
| **MIP-1a** | 76.2 (55.0-127) | 64.5 (56.4-92.7) | 77.1 (175-59.7) | | | | 88.7 (53.6-116) | 111 (62.1-215) | 64.0 (56.0-118) | 80.6 (43.3-99.7) | 63.5 (47.7-99.0) | 57.4 (48.3-84.5) |
| **TNF-b** | 10.0 (10.0-10.0) | 10.0 (10.0-10.0) | 10.0 (10.0-10.0) | | | | 10.0 (10.0-10.0) | 10.0 (10.0-10.0) | 10.0 (10.0-10.0) | 10.0 (10.0-10.0) | 10.0 (10.0-10.0) | 10.0 (10.0-10.0) |
| **GROa** | 3.46 (2.45-4.56) | 3.16 (2.07-4.25) | 2.50 (2.24-3.94) | | | | 2.48 (1.43-3.70) | 2.35 (2.10-3.32) | 2.53 (1.43-3.51) | 2.13 (1.43-3.54) *a* | 1.90 (1.09-3.44) | 1.66 (0.92-4.72) |
| **IL-1a** | 3.79 (1.52-12.7) | 1.63 (1.52-4.42) | 3.94 (1.52-5.92) | | | | 1.52 (1.52-3.04) | 1.52 (1.52-1.85) | 1.80 (1.52-5.84) | 5.35 (1.52-7.87) | 1.94 1.52-3.41) | 3.02 (1.52-4.16) |
| **IL-15** | 2.67 (2.50-5.90) | 3.67 (2.50-7.54) | 2.50 (2.50-7.01) | | | | 2.50 (2.50-9.62) | 2.92 (2.50-7.24) | 2.93 (2.50-8.73) | 3.07 (2.50-6.76) | 2.75 (2.50-5.79) | 2.50 (2.50-3.47) |
| **IL-22** | 216 (86.7-339) | 238 (151-285) | 319 (241-379) | | | | 205 (76.6-442) | 293 (252-350) | 323 (180-451) | 76.6 (76.6-124) | 76.6 (76.6-190) | 93.7 (76.6-155) |

Median (IQR). For between-arm comparisons (ANOVA) at each time point: * P≤0.05; ** P≤0.01; *** P≤0.001. For within-arm comparison (sign test) to baseline values: *a* P≤0.05; *b* P≤0.01; *c* P≤0.001

**Supplementary Table 8: T cell functional indices.**

| | Enrolment | | | Day 84 | | |
|---|---|---|---|---|---|---|
| | S-RUTF | F-RUTF | FFO-RUTF | S-RUTF | F-RUTF | FFO-RUTF |
| **CD4: CD38+ HLA-DR+** | 11.9 (9.44-17.6) | 9.25 (7.00-16.0) | 11.4 (8.49-14.2) | 12.3 (11.2-14.2) | 14.5 (10.3-18.6) | 17.3 (8.07-20.5) |
| **CD4: CD38+ HLA-DR+ PD-1+** | 5.49 (3.77-7.82) | 4.64 (3.22-6.93) | 5.67 (3.33-6.90) | 6.02 (4.33-7.49) | 6.36 (4.60-8.26) | 6.70 (3.36-10.8) |
| **CD8: CD38+ HLA-DR+** | 46.6 (38.6-54.9) | 48.9 (34.5-60.0) | 49.2 (24.6-65.0) | 47.4 (29.9-70.0) | 51.6 (40.7-62.0) | 51.6 (37.1-62.3) |
| **CD8: CD38+ HLA-DR+ PD-1+** | 23.5 (15.6-28.9) | 24.6 (11.3-36.1) | 18.6 (9.35-32.3) | 17.5 (11.1-28.7) | 18.2 (14.5-22.8) | 20.1 (16.6-22.7) |
| | | | | | | |
| **IFNg release: PHA** | 1017 (434-2086) | 722 (135-3090) | 273 (8-600) | 827 (325-3927) | 1127 (224-1526) | 970 (572-1504) |
| **IFNg release: PHA+IL-12** | 4510 (2802-7579) | 3338 (1724-11681) | 3569 (2132-5766) | 4525 (2050-12519) | 5207 (3156-9138) | 5109 (3520-8841) |
| **IFNg release: TT+IL-12** | 303 (69-765) | 233 (86-1225) | 203 (35-328) | 184 (48-489) | 83 (56-204) | 214 (69-965) |

Percentage co-expression of CD38 and HLA-DR; and CD38, HLA-DR and PD-1 on CD4+ or CD8+ T lymphocytes. Interferon-gamma release (with background subtraction) upon stimulation with phytohemagglutinin (PHA) +/- IL-12 or tetanus toxoid (TT) + IL-12 in pg/ml. Median (IQR). For between-arm comparisons (ANOVA) at each time point: * P≤0.05; ** P≤0.01; *** P≤0.001. For within-arm comparison (sign test) to baseline values: *a* P≤0.05; *b* P≤0.01; *c* P≤0.001
